# *PsicoCare*: a pilot randomized controlled trial testing a psychological intervention combining cognitive-behavioral treatment and positive psychology therapy in acute coronary syndrome patients

**DOI:** 10.3389/fpsyg.2024.1420137

**Published:** 2024-11-19

**Authors:** Inés Magán, Rosa Jurado-Barba, Guillermo Moreno, María Paz Ayán-Sanz, Juan Izquierdo-Garcia, Guido Corradi, Rocio Tello, Héctor Bueno

**Affiliations:** ^1^Facultad HM de Ciencias de la Salud de la Universidad Camilo José Cela - Villanueva de la Cañada, Madrid, Spain; ^2^Instituto de Investigación Sanitaria HM Hospitales, Madrid, Spain; ^3^Instituto de Investigación Hospital 12 de Octubre (Imas12), Madrid, Spain; ^4^Department of Cardiology, Hospital Universitario 12 de Octubre, Madrid, Spain; ^5^Facultad de Enfermería, Fisioterapia y Podología, Universidad Complutense de Madrid, Madrid, Spain; ^6^Department of Rehabilitation, Hospital Universitario 12 de Octubre, Madrid, Spain; ^7^Departamento de Psicología, Universidad Villanueva, Madrid, Spain; ^8^Multidisciplinary Translational Cardiovascular Research Group, Centro Nacional de Investigaciones Cardiovasculares (CNIC), Madrid, Spain; ^9^Facultad de Medicina, Universidad Complutense de Madrid, Madrid, Spain; ^10^Centro de Investigación Biomédica en Red Enfermedades Cardiovaculares (CIBERCV), Madrid, Spain

**Keywords:** cardiac rehabilitation, psychological intervention, positive psychology, cognitive-behavioral therapy, randomized controlled trial

## Abstract

**Background:**

Although psychological factors play a significant role in the onset and prognosis of acute coronary syndrome (ACS), psychological interventions (PIs) are rarely included in cardiac rehabilitation (CR) programs due to inconclusive evidence regarding specific intervention components and effect sizes. This study aimed to assess the efficacy of a PI based on cognitive-behavioral treatment (CBT) and positive psychology therapy (PPT) in improving psychological and clinical outcomes in patients with ACS.

**Methods:**

This *PsicoCare* trial was an open-label randomized controlled trial that compared a combined CBT and PPT-based PI (the *PsicoCare* program) with a standard CR program (control group). We recruited 87 ACS patients, and psychological outcomes, functional capacity, biochemical and anthropometric measures, and clinical outcomes were assessed at baseline, 2 months, and 9 months after the ACS event.

**Results:**

The *PsicoCare* group showed significant improvements in depression, anger traits, anger-in, and anger control-out compared to the control group. Additionally, the *PsicoCare* intervention was associated with the improved maintenance of cognitive function, social support, and spiritual coping styles, while the control group showed deterioration in these areas. Patients experiencing severe ACS showed significant improvement in personal strength and meaning as a result of the *PsicoCare* intervention. However, no significant effects were observed on anxiety, anger-out, emotion regulation skills, dispositional optimism, other personal strengths, or quality of life. Both groups demonstrated similar improvements in functional capacity and clinical outcomes.

**Conclusion:**

The study suggests that CBT and PPT-based PIs may offer additional benefits for ACS patients, particularly regarding their psychological health. Further larger trials are required to confirm these findings.

**Clinical trial registration:**

identifier, NCT05287061.

## 1 Introduction

Acute coronary syndromes (ACS) are characterized by a multifactorial nature influenced by classical cardiovascular (CV) risk factors such as sex, age, health status, body mass index, diabetes, and cholesterol levels, as well as lifestyle factors including smoking, exercise habits, and dietary choices. Additionally, psychological factors such as stress and negative emotions, including depression, anxiety, anger, and hostility, also play a significant role (Linden et al., 2007; Rozanski, 2014; Knuuti et al., 2020). Indeed, these psychological factors not only contribute to the onset of ACS but are also associated with poorer prognosis and an increased risk of recurrence (Nicholson et al., 2006; Chida and Steptoe, 2009; Roest et al., 2010; Russ et al., 2012).

Since the 90s, many well-designed studies testing the efficacy of different psychological interventions (PIs) for patients with ACS or CV disease have been conducted, as it has been thoroughly discussed in different meta-analyses and systematic reviews (Linden, 2000, 2013; Linden et al., 2007; Dickens et al., 2013; Rutledge et al., 2013; Richards et al., 2018; Magán et al., 2021, 2022). Specifically, PIs have shown benefits not only for psychological outcomes (depression, anxiety, stress, anger, and hostility) (Linden, 2000, 2013; Linden et al., 2007; Dickens et al., 2013; Rutledge et al., 2013; Richards et al., 2018; Magán et al., 2021) but also for biomedical and clinical outcomes (i.e., heart rate, total cholesterol, CV morbimortality or global mortality) (Linden et al., 2007; Rutledge et al., 2013; Richards et al., 2018; Magán et al., 2022).

These data are important because there is sufficient evidence supporting that patients with stress, anxiety and depression symptoms, or dysfunctional anger-hostility after suffering a cardiac event tend to have a worse prognosis, a higher risk of CV relapse or global morbimortality (Nicholson et al., 2006; Chida and Hamer, 2008; Roest et al., 2010). Although the mechanisms explaining the effects of PIs on CV health remain inconclusive, there may be two plausible pathways: a direct one that could increase cardiovascular risk by activating the central nervous and sympathetic systems, leading to dysfunctional CV reactivity and delayed recovery from physiological changes (e.g., elevated levels of glucocorticosteroids, cortisol, epinephrine, norepinephrine, heart rate, blood pressure, or inflammatory mediators) (Lovallo and Gerin, 2003; Schwartz et al., 2003; Hamer and Malan, 2010; Steptoe and Kivimäki, 2013; Rozanski, 2014; Wirtz and von Känel, 2017), and indirect mechanisms, as negative affect and stress, are often linked to unhealthy lifestyles and to a poor adherence to medication and medical recommendations (Rozanski, 2014).

Despite the potential efficacy of psychological interventions (PIs) on psychological and clinical outcomes (Magán et al., 2021, 2022), they are rarely included in usual cardiac rehabilitation (CR) programs (Poffley et al., 2017; Moghei et al., 2019), with exceptions (Abreu et al., 2019; Supervia et al., 2019). This could be explained by the fact that some variables relevant to treatment design remain inconclusive, such as the specific components to be included, the professional who should design and develop the PIs, or the duration of the intervention (Linden, 2013). Moreover, although PIs are beneficial, their effects are modest in size (Linden, 2000; Linden et al., 2007; Dickens et al., 2013; Rutledge et al., 2013; Richards et al., 2018).

Cognitive-behavioral treatment (CBT) has been empirically supported as the most effective type of PI for patients with ACS, especially if it is multi-component (including psychoeducation, relaxation techniques, problem-solving training, cognitive restructuring, stress, and anger management), individualized, and designed and applied by a health psychologist or a therapist specifically trained on mental health interventions (Linden, 2013).

However, CBT-based PIs for ACS patients have predominantly focused on changing negative emotions and lifestyle habits (Linden, 2000, 2013) rather than on enhancing positive psychological dimensions and wellbeing, despite its potential benefits in ACS patients (Huffman et al., 2016; Magán et al., 2021; Mohammadi et al., 2018; Sanjuan et al., 2016; Bolier et al., 2013; Nikrahan et al., 2016). Based on the positive psychology paradigm (Seligman et al., 2005), a new concept—cardiovascular positive health—has emerged (Labarthe et al., 2016), focusing on positive psychological factors such as dispositional optimism, positive emotions, life purpose, and life satisfaction, which have been empirically supported for their cardioprotective role (Boehm and Kubzansky, 2012; DuBois et al., 2015; Labarthe et al., 2016). Some PIs based on this paradigm have shown positive effects on cardiac rehabilitation patients (Bolier et al., 2013; Huffman et al., 2016; Nikrahan et al., 2016; Sanjuan et al., 2016; Mohammadi et al., 2018), and recent meta-analyses have concluded their benefits in improving life satisfaction, wellbeing, and reducing distress (Magán et al., 2021, 2022; Tönis et al., 2023).

PPT interventions can include only one component that focuses on positive thoughts and feelings, optimism, or gratitude. However, they are predominantly multicomponent. These interventions typically include practices aimed at developing gratitude, kindness, forgiveness, positive emotions, strengths, virtues, life purposes, and optimism, drawing on the proposals of Fredrikson, Seligman, and Fordyce. Although the evidence remains inconclusive, these positive dimensions may contribute to promoting CV health and mitigating the progression of CV disease through two pathways: a direct pathway, in which they influence biological processes associated with CV health (e.g., immune system function, low cortisol levels, CV reactivity, and heart rate function), and an indirect pathway, where they seem to foster the acquisition and maintenance of healthy lifestyles alongside a broader array of social and psychological CV protective factors (Steptoe et al., 2005; Labarthe et al., 2016; Rozanski et al., 2019).

Nevertheless, the approach of a combined CBT and positive psychology therapy (PPT)-based PI to improve psychological and clinical outcomes has not been tested so far.

*PsicoCare* is an open-label randomized controlled trial (RCT) aimed to assess the efficacy of a PI based on CBT and PPT principles, compared with a standard CR program (control group) in ACS patients. Specifically, the aim was to assess the benefits of the intervention in the following areas: (1) psychological factors—both negative ones (anxiety, depression, anger, coping, and emotion regulation) and positive psychological dimensions (psychological strengths, dispositional optimism, and quality of life), (2) functional capacity, and (3) biochemical and anthropometrical outcomes, as well as clinical outcomes of CV and global morbimortality. It was expected that the benefits of *PsicoCare* treatment would be significantly greater in these areas than in the control group.

## 2 Materials and methods

### 2.1 Participants

Participants were recruited from the Cardiology Department following an acute coronary event (myocardial infarction (MI) or unstable angina) before being referred to the CR program. The inclusion criteria were: (1) age ≥ 18 years, (2) hospitalization for an acute coronary event (MI or angina), and (3) referral to the hospital CR program. Exclusion criteria were a final diagnosis other than ACS, diagnosis of a major psychiatric disorder (these cases were referred to the hospital psychiatric service), and inability to follow the program for any reason (logistics, language barriers, and so on).

As this is pilot research and in line with Lakens (2022) conclusions, it was decided to optimize all available research and hospital resources. Therefore, the sample size criteria were based on including the maximum number of patients available during the study period. Thus, no *a priori* power analysis was conducted.

All patients who met the inclusion criteria without any exclusion criteria were invited to participate in the study by either the cardiologist or the nurse (HB or GM), and after signing the written informed consent, they were randomly assigned to the treatment group (*PsicoCare* program) or to the control group (usual care CR program) by the nursing staff (GM). A simple randomization method was used to minimize potential selection and allocation biases across groups. Both the professionals and the patients were aware of the assigned treatment branch, as blinding was not possible. Once patient consent was obtained and randomization to the intervention or control group occurred, trained health psychologists were notified to develop the psychological baseline assessment protocol and carry out the intervention.

Sociodemographic data (age, sex, educational level, marital status, and employment status), health status (including CV disease and other comorbidities), and ACS event characteristics (ACS type, the presence of chronic ACS, percutaneous intervention, bypass and complete revascularization, stroke, and bleeding during hospitalization, and Killip index) were recorded by cardiologists and cardiology nurses during the baseline clinical and medical assessment at the time of hospitalization.

**Figure 1:**
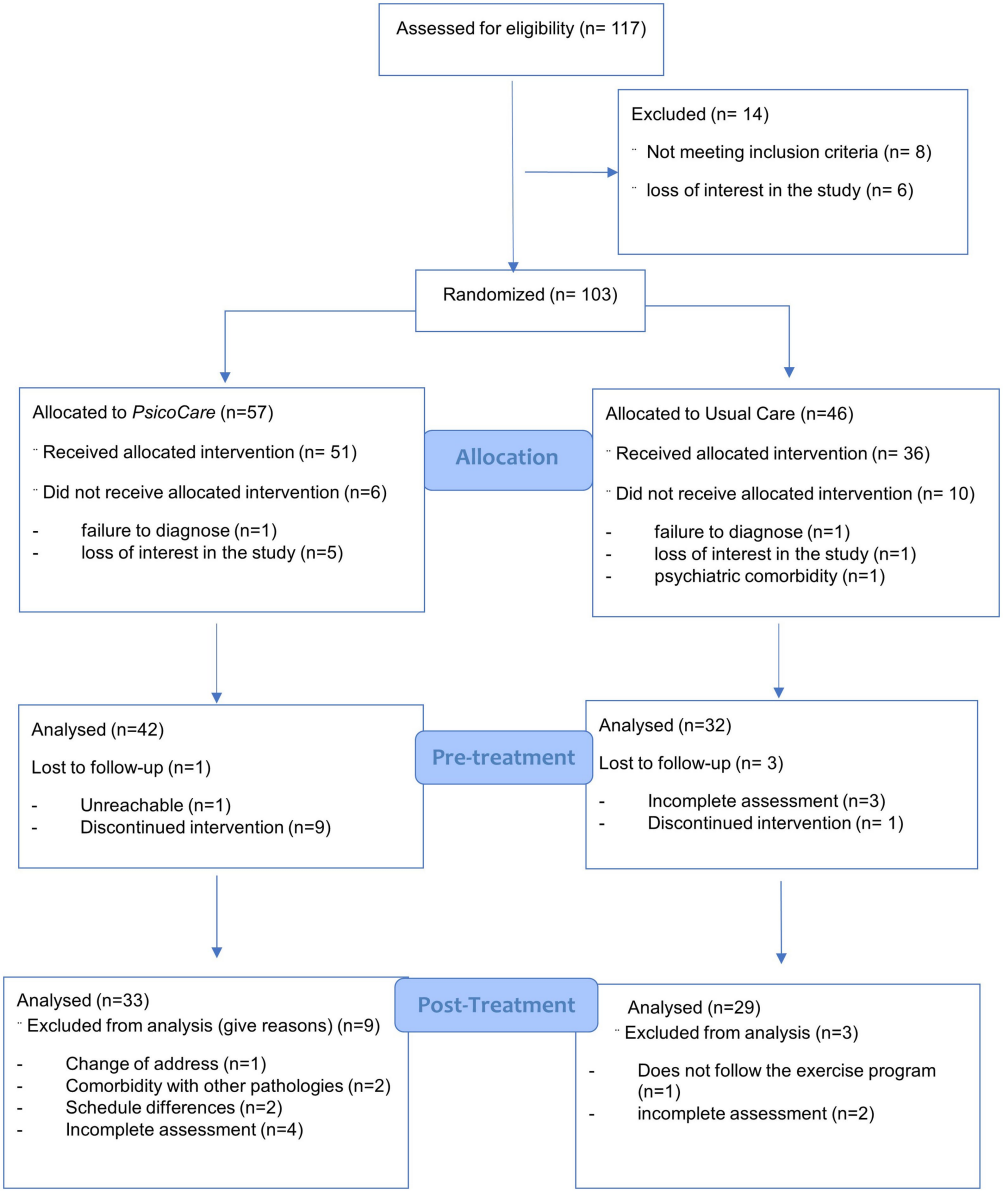
Flowchart diagram.

Initially, 117 patients were enrolled in the study, 103 of whom were randomized into two independent groups (see flowchart in Figure 1), and finally, 87 began the trial: *PsicoCare* (*n* = 51) and control group (*n* = 36). Baseline characteristics for both groups are presented in Table 1. Overall, both groups were similar, with a majority of male patients and an average age of 57 in the *PsicoCare* group and 60 years old in the control group. Most patients had a primary or secondary education level (53.8% in the *PsicoCare* group vs. 62.6% in the control group) and were working or retired due to age (65.4% vs. 70.2%).

**Table 1:** Differences between groups on sociodemographic, health status, ACS characterization, biochemical and anthropometrical data, and psychological outcomes at baseline assessment during hospitalization (*N* = 87).

Variables	Groups		Statistics	
	Control (*n* = 36)	PsicoCare (*n* = 51)	ᵪ^2^/t	*p*
Sex [% (*n*)]				
Men	91.8 % (33)	78.4 % (40)	1.97	0.160
Women	8.3 % (3)	21.1 % (11)		
Age [years; M (SD)]	59.90 (9.02)	57.20 (11.30)	1.14	0.256
Origin [% (*n*)]				
Caucasian	86.1 % (31)	76.9 % (40)	0.84	0.660
Latin	5.4 % (2)	5.7 % (3)		
Arab	0 % (0)	2 % (1)		
Marital status [% (*n*)]				
Single	19.0 % (7)	16.4 % (8)	7.19	0.210
Married	54.0 % (20)	50.0 % (26)		
Divorced	8.1 % (3)	3.8 % (2)		
Widowed	5.4 % (2)	3.8 % (2)		
Coupled	0 % (0)	11.5 % (6)		
Educational level [% (*n*)]				
Without education	5.4 % (2)	5.7 % (3)	5.71	0.225
Primary	35.1 % (13)	28,8 % (15)		
Secondary	37.5 % (15)	25.0 % (13)		
Graduate	5.4 % (2)	17.3 % (9)		
Postgraduate	2.7 % (1)	7.6 % (4)		
Working status [% (*n*)]				
Working	37.8 % (14)	42.3 % (22)	10.2	0.420
Housekeeper	2.7 % (1)	7.6 % (4)		
Unemployed	8.1 % (3)	7.6 % (4)		
Retired due to health issues or incapacity	13.5 % (5)	7.6 % (4)		
Retired due to age	32.4 % (12)	23.1% (12)		
Socioeconomic status [% (*n*)]				
Low	21.6% (8)	16.4 % (8)	4.29	0.232
Medium-low	27.0% (10)	21.1 % (11)		
Medium	46.0% (17)	40.3 % (21)		
Medium-high	0% (0)	9.6 % (5)		
High	0% (0)	0% (0)		
Health status [% (*n*)]				
Hypertension	46.0% (17)	46.0% (24)	0.03	0.085
Insulin-dependent diabetes mellitus	8.0% (3)	2.0% (1)	1.89	0.169
Non-insulin-dependent diabetes mellitus	16.0 % (6)	12.0% (6)	0.38	0.538
Hyperlipidemia	59.4 % (22)	50.0 % (26)	0.57	0.460
Moderate liver illness	0% (0)	2.0% (1)	0.74	0.389
Severe liver illness	2.7% (1)	0% (0)	1.41	0.236
Peptic ulcer	2.7 % (1)	9.8% (5)	1.74	0.187
Cancer within the last 5 years	2.7% (1)	9.8% (5)	1.74	0.187
CV comorbidities [% (*n*)]				
Stroke	5.4% (2)	2% (1)	0.78	0.375
Myocardial infarction	19.4% (7)	17.3% (9)	0.05	0.816
Coronary percutaneous interventions	19.4% (7)	11.7% (6)	0.86	0.353
Peripheral arterial disease	5.5% (2)	7.8% (4)	0.18	0.667
Atrial fibrillation	0% (0)	1.9% (1)	0.74	0.389
Valvular prosthesis	0% (0)	1.9% (1)	0.74	0.389
Smoking habit [% (*n*)]				
Yes	35.1 % (13)	25.0 % (13)	0.66	0.718
No	35.1 % (13)	30.7 % (16)		
Past-smoker	24.3 % (9)	21.1 % (11)		
CAD event characteristics [% (*n*)]				
ACS type				
STEMI	47.2% (17)	56.8% (29)	0.97	0.323
NSTEMI	52.7% (19)	41.1% (21)		
Obstructive coronary artery disease	91% (33)	92.1 % (47)	0.17	0.675
Percutaneous intervention	86% (31)	90.0 % (46)	0.77	0.379
Failed percutaneous intervention	0% (0)	5.8% (3)	2.24	0.135
Complete coronary revascularisation	55.5% (20)	66.6% (34)	1.33	0.248
Stroke during hospitalization	0% (0)	0% (0)	-	-
Serious hemorrhage during hospitalization	0% (0)	0% (0)	-	-
Biochemical and anthropometric data [M (SD)]				
BMI	28.7 (4.3)	29.5 (4.5)	0.78	0.437
SBP (mm Hg)	124.8 (24.7)	122.3 (22.1)	0.47	0.640
DBP (mm HG)	73.8 (12.2)	70.8 (11.7)	1.15	0.252
Heart rate (beats per minute)	75.8 (18.1)	74.9 (14.3)	0.25	0.802
LDL cholesterol (mg/dl)	86.3 (42.5)	103.6 (39.2)	1.89	0.063
Glycated hemoglobin (%)	6.2 (1.5)	5.7 (0.6)	1.84	0.073
Peak troponin value	2861.4 (4044.5)	2,111 (2727.7)	0.95	0.343
Killip class	1.3 (0.8)	1.2 (0.8)	0.31	0.752
Left ventricular ejection fraction (LVEF)	54.2 (8.9)	53.5 (9.0)	0.34	0.734
Anxiety [HADS-A; M (SD)]	8.5 (3.5)	9.2 (2.9)	0.96	0.320
Depression [HADS-D; M (SD)]	4.1 (3.1)	5.3 (3.5)	1.68	0.097
Quality of life [SF-12; M (SD)]				
Physical	41.7 (7.9)	42.4 (6.9)	0.48	0.628
Mental	47.0 (5.7)	45.7 (5.9)	1.02	0.311

There were no statistically significant differences between the groups in terms of risk factors, prior CV history, ACS characteristics, or treatment with percutaneous intervention.

### 2.2 Measures and outcomes

Given the specific objectives to test the efficacy of *PsicoCare* vs. standard treatment, all obtained measures were considered primary outcomes. The instruments and variables used are specified below.

*Psychological outcomes: as described above, psychological outcomes were measured at times 1, 2, and 3* (Supplementary Figure S1).

- Anxiety and depressive symptomatology: Hospital Anxiety and Depression Scale (HADS) (Zigmond and Snaith, 1983; Tejero et al., 1986).
- Anger facets: State–Trait Anger Expression Inventory 2-STAXI 2 anger trait, anger expression, and anger control scales (Spielberger et al., 2001; Spielberger, 1999).
- Coping skills: Coping Questionnaire short form, COPE 28 (Carver, 1997; Morán et al., 2010).
- Emotion regulation skills: Difficulties in Emotion Regulation Scale (DERS) (Gratz and Roemer, 2004; Hervás and Jódar, 2008)
- Dispositional optimism: Life Orientation Test revised LOT-R (Scheier et al., 1994; Otero et al., 1998).
- Personal strengths: Positive Emotion, Engagement, Relationships, Meaning, and Accomplishment Questionnaire (PERMA) (Seligman, 2011).
- Quality of life: Questionnaire SF-12 (Ware et al., 1996; Vilagut et al., 2008)

*Functional capacity outcomes* were measured using two stress tests conducted by a specialized physiotherapist and physician at times 2 and 3, and included the following parameters: stress test time, METS, resting and maximal blood pressure and heart rate levels, the clinical and electrical significance of the stress test, and the presence of arrhythmia during the test.

*Biochemical and anthropometrical outcomes* were measured at times 1 and 3 by a cardiologist, including blood pressure, heart rate, body mass index, LDL cholesterol, and glycated hemoglobin were considered.

*Clinical outcomes* were recorded from electronic health records 9 months after the ACS event (posttreatment assessment), at time 3. Non-fatal MI, non-fatal stroke, hospitalization for CV cause, all-cause mortality, and CV mortality were the outcomes considered in this study.

### 2.3 Procedure

#### 2.3.1 Study protocol

Once informed consent was obtained, participants were randomized and assigned to one of two arms: the experimental group (*PsicoCare*) and the control group (usual care CR program). The trial protocol is described in detail in Supplemental Material 1.

All patients were assessed at three different times (Supplementary Figure S1): (1) Time 1, initial baseline assessment (biochemical, anthropometric, clinical, and psychological outcomes) conducted during the first 48 h after the cardiac event; (2) Time 2, pretreatment assessment (psychological and functional capacity outcomes), 2 months after the event (with a + - 2 weeks before or after deviation in order to have enough participants for each condition), and (3) Time 3, posttreatment assessment (clinical, psychological and functional capacity), 9 months after the event. Specific outcomes are described below.

*PsicoCare* patients (experimental group) received a short early intervention called *Health Pills during hospitalization*. After a period of 2 months, the *PsicoCare* group started the first phase of the group intervention program (Phase 1), followed by the standard CR program, similar to the control group (Supplementary Figure S1). *The PsicoCare* group then completed Phase 2, which consisted of the final three group sessions. Supplementary Figure S1 and Table 1 describe the *PsicoCare* treatment procedure and protocol.

Patients allocated to the control group received only the standard CR program, which included a group education program and a physical activity training program (Supplementary Figure S1). They were assessed at the same three-time points as the *PsicoCare* group.

#### 2.3.2 Intervention

As described above, the experimental *PsicoCare* program was implemented during the final phase of hospitalization and continued after discharge, comparing its effects with the standard CR program at three different time points.

Briefly, the *PsicoCare* program was a PI based on CBT and PPT principles designed by specialized health psychologists and structured in two parts: (1) a short early intervention called Health Pills, consisting of two individual sessions developed by trained health psychologists.

The first session took place within the first 48 h after hospitalization, followed by the second 1 week after discharge. These sessions used motivational interviewing and emotional discharge techniques to equip patients with coping strategies and emotional management skills (2). The second part of the intervention consisted of six weekly group sessions developed by two trained health psychologists (Supplementary Table S1), starting 2 months after discharge. The first three sessions, based on CBT and Seligman’s exercises (Seligman et al., 2005), aimed to (1) promote and consolidate personal growth after the CV event, (2) improve adaptive coping style, and (3) develop skills to manage negative emotions while fostering positive emotions. After completing the first three sessions, the patients allocated to the intervention arm completed the hospital CR program (see below). Then, the final three sessions of *PsicoCare* were developed to consolidate the skills learned, set life goals, and foster personal growth. The specific objectives and details of the *PsicoCare* sessions are described in Supplementary Table S1.

The control group received a conventional hospital CR program consisting of a clinical and educational program focused on optimizing secondary prevention therapies (lifestyle and medication) delivered by a team including cardiologists and specialist nurses and an 8-week group exercise program developed by a physical rehabilitation physician and physiotherapist. The education program provided knowledge about cardiac disease, medication, and lifestyle management. The exercise training program aimed to restore patients’ maximum physical capacity after the cardiac event and improve their social, family, and occupational quality of life, thereby reducing future cardiac morbidity and mortality.

### 2.4 Design and statistical analysis

This study was conducted according to a randomized clinical trial (RCT) design in the Cardiology Department of Hospital Universitario 12 de Octubre, Madrid (Spain), between the 1st of July 2017 and the 31st of October 2018 (registered at Clinical Trials, NCT05287061). Within the first 48 h after the cardiac event and during hospitalization, the enrollment proposal was made to those who *a priori* should meet the inclusion criteria. Once they accepted and the informed consent was signed, the baseline assessment and the first *Health Pills* session were developed (see Supplementary Figure S1). This RCT was reported according to CONSORT criteria (Schulz et al., 2010; Montgomery et al., 2018). This project was approved by the Ethics Committee of Hospital 12 de Octubre (17/171).

A 2×2 (group x assessment time) repeated measures experimental design was used in this RCT. χ^2^ and student’s t-test were used to assess the equivalence between groups for qualitative and quantitative variables at baseline assessment (Time 1). A linear mixed model analysis was performed to assess the effectiveness of the *PsicoCare* treatment condition compared to the control group. Linear mixed-effects models are the most suitable analyses to test the efficacy and treatment effects, as this statistical method simultaneously accounts for both between-subject and within-subject effects of the independent variables (Baayen et al., 2008), reducing type-I error and enhancing generalizability of the results. The database and statistical analysis were carried out using R statistical software.

Satterthwaite’s method for calculating degrees of freedom was used to correct post-hoc tests for multiple comparisons and *p*-values using the multivariate *t-*distribution adjustment (see Supplementary material S2). Two models were developed for each outcome of interest, including momentary assessment and group membership as an interaction to assess the effect of *PsicoCare* improvement. All models included participants as a random effect to capture the individual variability. The first model (Model 1) was set up to examine the effect of the condition (*PsicoCare* condition vs. control group), and the second model (Model 2) was set up to examine the effect of different variables of interest. The extended model included the triple interaction group x time x ACS severity. The control variables were sex, age, socioeconomic status, and ACS severity. To check whether our data were suitable for developing a linear mixed model analysis, model fit indices were calculated: Akaike’s Information Criteria (AIC), pseudo-R^2^ marginal (accounts for the fixed effect) and conditional (accounts for the whole model), as well as intra-class correlation coefficients (ICC) for the models. All model fit indices indicated our data were suitable for developing a linear mixed model analysis for checking *Health Pills* efficacy, as well as the *PsicoCare* efficacy program on psychological, physical function, and biochemical and anthropometric outcomes (see Supplementary Tables S2–S5), showing a suitable intra-class correlation and explained variance. Model 2 showed better comparative goodness of fit index (using the AIC) for *Health Pills* efficacy analysis (Supplementary Table S2), but also for *PsicoCare* efficacy analysis on psychological, physical function, and biochemical and anthropometric outcomes (see Supplementary Tables S3–S5, respectively, for details).

## 3 Results

### 3.1 Efficacy of health pills on psychological outcomes

Anxiety, depression, and quality of life were measured before the starting of the *Health Pills* phase at baseline (Time 1) and 2 months after this intervention phase. Despite numerically lower values in anxiety, depression, and physical and mental quality of life measures between both groups, these were not statistically significant differences in the simple (Model 1) or the complex model (Model 2), adjusted for sociodemographic variables and ACS type (Supplementary Tables S2, S6).

### 3.2 Efficacy of the *PsicoCare* program on psychological outcomes

**Figure 2:**
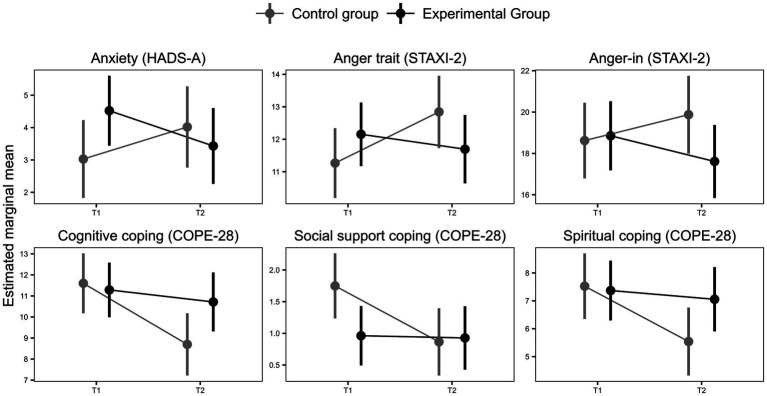
*PsicoCare* and control group changes between pre- and post-treatment assessment on anxiety (HADS-D), anger-trait (STAXI 2), anger-in (STAXI 2), cognitive, social support, and spiritual coping (COPE 28).

The *PsicoCare* program improved some of the negative psychological outcomes between pretreatment and post-treatment assessment (Time 2 and Time 3; see Table 2 and Figure 2), specifically depressive symptoms (EMM_T2-T3_ 1.10 ± 0.62 vs. −1.00 ± 0.68; b, −2.08; 95%CI, −3.90 to -0.26, *β =* −0.61, *p* = 0.03); anger trait (EMM_T2-T3_ = 1.24 ± 0.75 vs. 0.48 ± 0.52; b, -2.50; 95%CI, −4.65 to −0.34; *β =* −0.48) and anger-in (EMM_T2-T3_, −1.25 ± 0.80 vs. 1.58 ± 0.55; b = −2.03; 95%CI, −3.52 to −0.55; *β =* −0.65, *p* < 0.01). When sociodemographic variables and ACS type were controlled in Model 2 (Supplementary Table S3), both groups showed higher scores on control-out and control-in anger dimensions. The *PsicoCare* group showed a significant improvement compared to the control group in anger control-out (EMM_T2-T3_ = 1.35, SE = 0.65 vs. 0.28, SE = 0.64; *b* = −3.20, 95% CI, −5.90 to −0.51, *β =* −0.79, *p* = 0.021) due to the effect of the intervention, while the benefit on anger control-in was significantly higher in the control group (EMM_T2-T3_ = −2.00, SE = 0.85; b = −3.70, 95% CI, −7.21 to -0.19, *β =* −0.82, *p* < = 0.0104) than in the *PsicoCare* group (EMM_T2-T3_ = -1.16, SE = 0.84). There was not any significant effect found for either anxiety or anger-out expression style (Table 2).

**Table 2:** *PsicoCare*: psychological outcomes descriptive statistics and change score between pre and post-treatment assessment.

Measure	Control group [M (SD)]			PsicoCare group [M (SD)]		
	Pre-treatment (T2)	Post-treatment (T3)	Change score (Δ)	Pre-treatment (T2)	Post-treatment (T3)	Change score (Δ)
Anxiety (HADS-A)	7.3 (3.5)	8.5 (4.4)	−1.0 (3.1)	8.1 (3.2)	7.7 (2.7)	0.4 (3.2)
Depression (HADS-D)	3.0 (3.0)	3.9 (3.8)	−1.1 (3.1)	4.5 (3.5)	3.4 (3.3)	1.0 (3.9)
Anger (STAXI 2)						
Anger-trait	18.8 (5.9)	19.7 (5.8)	−1.5 (4.2)	18.9 (5.2)	17.5 (3.9)	1.2 (4.2)
Anger-out	10.3 (3.3)	10.5 (3.3)	0.1 (2.6)	10.3 (3.0)	10.0 (2.9)	0.7 (3.0)
Anger-in	11.3 (2.8)	12.7 (3.2)	−1.7 (3.1)	12.2 (2.9)	11.8 (3.5)	0.4 (2.8)
Anger control-out	16.2 (4.4)	17.3 (3.9)	−1.4 (4.1)	17.7 (3.9)	18.5 (3.7)	−0.5 (2.7)
Anger control-in	13.7 (4.6)	15.6 (4.3)	−2.1 (5.5)	14.2 (4.5)	15.2 (4.4)	−1.4 (3.8)
Coping (COPE 28)						
Cognitive	11.7 (3.2)	8.8 (5.6)	2.9 (4.6)	11.4 (3.6)	10.7 (3.9)	0.4 (3.7)
Avoidance	7.8 (4.9)	6.6 (5.4)	0.9 (4.8)	7.5 (3.2)	6.9 (3.3)	0.4 (3.7)
Social support	7.6 (3.2)	5.6 (3.6)	2.0 (3.4)	7.5 (3.3)	7.1 (3.4)	0.3 (3.0)
Spiritual	1.8 (1.9)	0.8 (1.5)	0.8 (1.4)	1.0 (1.1)	0.9 (1.4)	0.03 (1.2)
Emotion regulation (DERS)	54.0 (19.6)	57.7 (16.6)	−5.7 (16.9)	56.0 (16.7)	54.3 (17.2)	−0.3 (13.8)
Lack of emotional attention	10.8 (3.6)	10.9 (2.2)	−0.1 (3.5)	9.8 (3.9)	11.0 (2.2)	−1.2 (3.2)
Emotional confusion	8.1 (3.0)	8.2 (3.0)	−0.1 (3.0)	7.4 (3.1)	7.2 (3.0)	−0.2 (1.7)
Emotional rejection	12.6 (7.3)	13.8 (5.8)	−2.2 (5.7)	13.9 (5.8)	12.5 (6.7)	0.5 (5.0)
Emotional lack of control	14.5 (6.5)	16.1 (6.4)	−2.4 (6.4)	15.4 (6.4)	14.8 (6.4)	−0.2 (5.8)
Emotional life interference	8.0 (3.0)	8.7 (2.8)	−0.8 (3.7)	9.5 (3.9)	8.8 (3.4)	0.7 (3.7)
Dispositional optimism (LOT-R)	15.4 (3.6)	14.5 (4.4)	1.5 (3.6)	14.8 (3.8)	14.7 (4.8)	0.3 (3.5)
Psychological Strengths (PERMA)	7.6 (1.2)	7.3 (1.7)	0.1 (1.5)	7.0 (1.6)	7.2 (1.8)	−0.2 (1.7)
Achievement	7.5 (1.4)	7.0 (1.8)	0.2 (1.6)	6.5 (1.7)	6.9 (1.7)	−0.3 (1.6)
Engagement	7.6 (1.5)	7.4 (1.8)	0.2 (1.7)	7.3 (1.7)	7.3 (1.9)	0.1 (1.9)
Meaning	7.6 (1.3)	7.2 (1.8)	0.2 (1.7)	6.9 (1.9)	7.3 (1.9)	−0.27 (1.7)
Positive emotions	7.6 (1.6)	7.3 (2.0)	0.1 (1.9)	6.7 (1.9)	7.2 (2.0)	−0.38 (2.0)
Social relationships	7.9 (1.4)	7.8 (1.8)	0.11 (1.6)	7.4 (1.9)	7.5 (2.1)	−0.03 (1.9)
Quality of life (SF12)						
Physical	41.6 (6.5)	40.9 (7.4)	1.1 (7.3)	41.8 (6.6)	43.1 (5.8)	−1.3 (7.3)
Mental	47.3 (5.6)	47.7 (5.4)	−1.3 (6.5)	46.9 (5.1)	49.3 (5.4)	−2.8 (7.5)

Regarding coping styles, some modest benefits from the *PsicoCare* intervention emerged when compared to the standard CR program (Table 2, Figure 2, and Supplementary Table S3). The *PsicoCare* group maintained adequate cognitive coping skills (EMM_T2-T3_ = 2.90, SE = 0.77; *b* = 2.33, 95% CI, 0.26 to 4.4, *β =* 0.56, *p* = 0.032771), social support (EMM_T2-T3_ = 0.31, SE = 0.56; *b* = 1.67, 95% CI, 0.05 to 3.29, *β =* 0.49, *p* = 0.04), and spiritual coping skills (EMM_T2-T3_ = 0.04, SE = 0.24; *b* = 0.85, 95% CI, 0.18 to 1.52, *β =* 0.56, *p* = 0.013), whereas the control group experienced a decline in these areas (EMM_T2-T3_ = 0.57, SE = 0.72; EMM_T2-T3_ = 1.98, SE = 0.60 and EMM_T2-T3_ = 0.88, SE = 0.25, for cognitive, social support, and spiritual coping, respectively).

Furthermore, Model 2, which controlled for sociodemographic factors and ACS severity, showed a similar pattern but revealed stronger results for spiritual coping when comparing the *PsicoCare* and control groups (EMM_T2-T3_ = -0.05, SE = 0.24 vs. EMM_T2-T3_ = 0.91, SE = 0.25; *b* = 1.64, 95% CI, 0.6 to 2.68, *β =* 1.05, *p* = 0.003).

Although there were no significant benefits related to emotion regulation skills in Model 1 (Table 2 and Supplementary Table S3), Model 2 showed a significant effect of the intervention (*b* = 3.03, 95% CI, 0.28 to 5.78, *β =* 0.95, *p* = 0.031), predicting an increase in the lack of emotional attention regulation skills in the *PsicoCare* group compared to the control group (EMM_T2-T3_ = −1.15, SE = 0.65 vs. EMM_T2-T3_ = 0.07, SE = 0.66).

Finally, in relation to positive dimensions and quality of life, Model 2 revealed, after controlling for sociodemographic factors and ACS severity effect, a significant triple interaction (time x group x ACS type), showing patients who had suffered the most severe ACS event (STEMI) in the *PsicoCare* group compared to control group significantly improved the meaning strength dimension (EMM_T2-T3_ = 0.77 ± 0.62 vs. EMM_T2-T3_ = −1.16 ± 0.63; b, 1.94; 95%CI, 0.15 to 3.73; *β* = 1,12, *p* = 0.03) on dispositional optimism or the rest of psychological strengths or quality of life (Supplementary Table S3).

### 3.3 Efficacy of the *PsicoCare* program on functional, biochemical, anthropometric, and clinical outcomes

The *PsicoCare* group showed no benefit in any of the functional measured outcomes (Table 3 and Supplementary Table S4) biochemical and anthropometric outcomes (Table 3 and Supplementary Table S5).

**Table 3:** *PsicoCare*: ergometry physical outcomes, biochemical and anthropometric descriptive statistics, and change score between pre and post-treatment assessment.

	Control group [% (n) / M (SD)]			PsicoCare group [% (n) / M (SD)]		
Measure	Pre-treatment (T2)	Post-treatment (T3)	Change score (T4)	Pre-treatment (T2)	Post-treatment (T3)	Change score (T4)
Ergometry physical outcomes						
METS	10.1 (3.8)	11.2 (4.1)	−1.2 (2.9)	9.6 (3.9)	10.9 (4.0)	−1.7 (3.5)
Total ergometry time	7.7 (3.5)	8.9 (3.7)	−1.54 (2.2)	7.7 (3.3)	9.5 (4.5)	1.51 (3.55)
Heart rate						
Maximum	131.2 (22.2)	134.1 (19.8)	−4.0 (15.6)	128.3 (37.2)	132.4 (18.6)	−10.1 (42.3)
Resting	68.1 (16.7)	64.5 (12.8)	2.3 (15.0)	67.9 (21.5)	67.9 (17.4)	2.4 (21.1)
Systolic blood pressure (mmHg)						
Maximum	161.7 (27.0)	173.4 (30.6)	−9.2 (22.2)	146.9 (41.4)	157.5 (23.0)	−9.5 (39.7)
Resting	115.6 (19.6)	123.8 (25.5)	−7.2 (30.4)	108.1 (32.1)	112.2 (17.3)	−5.0 (28.0)
Diastolic blood pressure (mmHg)						
Maximum	77.7 (12.1)	80.2 (14.7)	0.0 (13.1)	73.6 (22.3)	75.0 (13.0)	−1.4 (22.6)
Resting	70.6 (9.7)	77.5 (12.0)	−5.8 (13.0)	68.6 (20.2)	68.8 (10.0)	−0.6 (20.8)
Clinical response						
Clinical significance	5% (2)	5% (2)	0% (0)	8% (4)	4% (2)	4% (2)
Electrical significance	15% (6)	12% (5)	3% (1)	14% (7)	6% (3)	8% (4)
Arrhythmia presence during ergometry	2% (1)	7% (3)	5% (−2)	6% (3)	4% (2)	2% (1)
Biochemical and anthropometrical outcomes						
Heart rate	75.8 (17.9)	62.1 (11.4)	11.93 (17.09)	75.0 (14.5)	68.5 (13.1)	7.48 (17.1)
Systolic blood pressure (mmHg)	121.3 (24.7)	138.5 (77.0)	−16.95 (80.61)	121.7 (21.1)	120.1 (10.0)	2.46 (18.5)
Diastolic blood pressure (mmHg)	72.5 (13.3)	75.3 (8.9)	−2.0 (15.44)	71.4 (12.7)	75.4 (9.4)	−3.83 (13.3)
LDL-cholesterol (mg/dl)	89.7 (42.6)	61.5 (24.0)	25.14 (51.97)	103.7 (38.1)	60.1 (18.1)	39.79 (40.6)
Glycated hemoglobin	6.2 (1.5)	6.5 (2.0)	−0.21 (1.45)	5.6 (0.6)	5.8 (0.5)	−0.03 (0.5)
BMI	28.3 (3.9)	33.5 (18.4)	−4.0 (18.93)	29.8 (4.6)	34.9 (17.6)	−4.05 (18.8)

Finally, regarding morbimortality outcomes (Table 4), although the control group required more new revascularization interventions, experienced a greater number of clinical outcomes, and necessitated more re-hospitalizations due to angina episodes, no statistical differences emerged between the groups.

**Table 4:** Differences in clinical outcomes during follow-up by group.

Variables	Group [% (n) / M (SD)]		*p*-value
	Control group (*n* = 36)	PsicoCare group (*n* = 51)	
Cardiovascular mortality	0% (0)	0% (0)	–
MI recurrence	0% (0)	0% (0)	–
Stroke recurrence	0% (0)	0% (0)	–
New revascularization needed	5% (2)	0% (0)	0.095
Number of clinical outcomes	0,16	0.08	0.471
New hospitalization because of angina	0.19	0.08	0.335

## 4 Discussion

*PsicoCare* evaluated the efficacy of a PI based on CBT and PPT principles on improving psychological factors, functional capacity, biochemical, anthropometric, and clinical outcomes, showing significant improvements in depression, anger trait, anger-in, and anger control-out compared with the usual care CR program and maintaining a more adaptive cognitive, social support, and spiritual coping styles. Patients who had experienced more severe ACS events showed greater personal strength of meaning improvement than the control group. However, there were no significant effects on anxiety, anger-out, avoidance coping, emotion regulation skills, dispositional optimism, or other personal strengths such as achievement, engagement, positive emotions, social relationships, or quality of life. The control group only showed a significant improvement in anger control and emotion regulation skills due to a lack of attention to emotions. Both groups achieved similar benefits in functional capacity measures and biochemical, anthropometric, and clinical outcomes.

The *PsicoCare* group intervention significantly improved depressive symptoms, supporting different meta-analyses (Linden et al., 2007; Dickens et al., 2013; Rutledge et al., 2013; Richards et al., 2018; Magán et al., 2021) and narrative reviews (Linden, 2000, 2013). However, in contrast with previous research (Michalsen et al., 2005; Richards et al., 2018), *PsicoCare* also promoted a more adaptive pattern of anger, not only reducing anger traits and anger-in but also promoting specific strategies for managing the experience of anger based on external cues (i.e., taking time-out to relax and reassess the situation to cope with it). The benefits observed for depression and certain dimensions of anger are likely due to the specific components of the *PsicoCare* treatment aimed at managing and coping with negative emotions following an ACS event. These improvements are particularly relevant, as depression and anger are important psychological factors that contribute to increased CV risk (Nicholson et al., 2006; Chida and Steptoe, 2009; Rozanski, 2014; Tully et al., 2015; Carney and Freedland, 2017). Depressive symptoms or dysfunctional anger increase the risk of morbidity and mortality after ACS, up to 90% with depression (Nicholson et al., 2006) and 24% with anger (Chida and Steptoe, 2009; Rozanski, 2014). For this reason, the need to screen and manage depression after an ACS is widely accepted (Fihn et al., 2014; Knuuti et al., 2020; McDonagh, et al., 2021), although no similar recommendations exist for anger (Michalsen et al., 2005).

Contrary to our expectations and prior evidence (Linden, 2000, 2013; Linden et al., 2007; Richards et al., 2018; Magán et al., 2021), there was no significant effect on anxiety, an important CV risk factor (Roest et al., 2010; Rozanski, 2014; Tully et al., 2015; Ossola et al., 2018). Both groups reduced their anxiety levels without difference between them. This unexpected result may be explained by a time-dependent attenuation of ACS-related anxiety and a dilution of the potential benefit of the intervention by the improvement produced by the standard CR program, which in our case included specific educational components for relaxation and stress management and breathing exercises.

The *PsicoCare* intervention significantly maintained a more functional cognitive, social support, and spiritual coping, while the control group deteriorated, probably explained by the specific components included in the experimental treatment. This is relevant because stress and dysfunctional coping after ACS have been shown to increase future morbidity and mortality risk by 72% (Arnold et al., 2012; Rozanski, 2014). Cognitive and social support coping styles are traditionally considered adaptive ways of dealing with problems and difficulties (Lazarus and Folkman, 1984; Carver, 2019), and both coping styles could contribute to a better adjustment to the new life situation after the coronary event, including all medical and lifestyle prescriptions. In contrast, there were no changes in emotion regulation strategies, perhaps because both groups already had adequate levels of these skills, with one exception: the *PsicoCare* group significantly reduced their attention to emotions. Although not paying enough attention to our emotions should be a dysfunctional strategy (Hervás and Jódar, 2008), the *PsicoCare* intervention was aimed at normalizing and accepting the experience of negative feelings, trying to facilitate coping, and managing them but not just focusing on the emotion without acting. Thus, it is possible that the *PsicoCare* intervention changed this pattern by redirecting the focus from negative emotions to accepting and normalizing these negative affective states.

Patients who had experienced the most severe ACS event showed a significant improvement in meaning personal strength due to the *PsicoCare* intervention, which could partially support the positive behavioral cardiology paradigm (Labarthe et al., 2016; Kubzansky et al., 2018; Steptoe, 2019; Boehm, 2021), what is coherent with the recent statement the American Heart Association has published, which outlines the key role of psychological wellbeing on CV health and encourages to include PIs aimed to improve psychological positive dimensions on preventive and CR programs (Levine et al., 2021). The *PsicoCare* intervention included specific components aimed at discovering patients’ values and purpose in life to enhance a meaningful personal life and personal growth after the event, as they are key components of psychological wellbeing, compliance, and health that could explain this intervention effect. The relevance of this finding is based on the CV-protective role of positive dimensions, especially meaning, values, and purpose in life, which appears to significantly reduce CV risk (Boehm and Kubzansky, 2012; Rozanski, 2014; Labarthe et al., 2016; Kubzansky et al., 2018; Boehm, 2021; Vos, 2021), especially because benefits were observed in all these more severe patients. However, there were no significant changes in the remaining psychological strengths (global, positive emotions, achievement, and social relationships) or in dispositional optimism. Although optimism is one of the most robust psychological cardioprotective factors (Rozanski, 2014; Rozanski et al., 2019), the lack of change in this dimension could be due to its dispositional state, which implies the characteristics of temporal stability and consistency between different situations. Therefore, perhaps the intervention should have focused more specifically and directly on this construct to achieve some changes and benefits on optimism.

CR programs aim not only to reduce CV risk and future morbidity and mortality but also to improve the quality of life in coronary patients (Fihn et al., 2014; Stenvall et al., 2017; Knuuti et al., 2020). However, the benefits of PIs in improving the quality of life of ACS McDonagh, et al. (2021) patients are weak (Richards et al., 2018), which is consistent with our results. *PsicoCare* did not improve the physical or mental dimensions of quality of life. The short time elapsed since the ACS event (9 months) could probably explain the lack of significant changes, as it should be in this period when patients are aware of the consequences of the CV event and try to adapt to their “new life.” Quality of life is a robust construct, so the changes in its dimensions are not usually perceived immediately. People need longer periods of time to become aware of changes in their quality of life (Anderson et al., 2016; Oldridge et al., 2022). Therefore, benefits on quality of life, at least on the mental dimensions, might have emerged if longer follow-ups had been developed.

Finally, both groups showed similar improvements in physical function and biomedical, anthropometric, and clinical outcomes, which is logical because both groups received the same usual care CR program (physical training and education program) and medical prescriptions. The difference between the two interventions was small, making our results more valuable. This was only a pilot study. By increasing our study’s statistical power and sample size, it is possible that some additional effects would emerge as significant. In addition, it is likely that the benefits on biochemical and clinical outcomes were not immediate and that psychological changes need to be long-lasting to positively affect biological outcomes, so longer follow-ups are probably required.

This research has some important limitations. One notable concern is the small sample size, which may explain the absence of statistically significant effects despite the presence of some differences. Additionally, the large number of outcomes considered could lead to random effects. However, the use of linear mixed-effect models helps control for this bias and enhances the generalizability of our results despite the limited sample size. Finally, patients are often required to attend multiple concurrent programs (educational, physical, psychological, etc.), which may interfere with their ability to participate fully and benefit from all interventions.

The PI whose efficacy was tested had two relevant novelties. First, it was based on two well-established psychological paradigms (CBT and PPT), based on the idea that, according to the positive behavioral cardiology paradigm, these two types of interventions working together would enhance the benefits and efficacy of PI in ACS patients. Second, it was structured in two phases: *Health Pills*, an early and brief intervention that was developed during the acute CV event phase, and the *PsicoCare* group intervention, which developed 2 months after the event. *Health Pills* resulted in additional benefits for patients in the experimental group compared to those in the control group. This may support the argument of Linden (2013), who suggested that the benefits of PIs were greater when the intervention started at least 2 months after the CV event. Being more resilient may make it easier to deal with the situation in a more adaptive style during these weeks without the need for a specific PI. Others, however, may benefit from a specific PI aimed at helping them to cope with their problem more functionally to improve various skills to cope with emotional distress and promote adherence, personal growth, wellbeing, and quality of life. Furthermore, it is possible that this early and brief psychological support did not produce the expected results because it was initiated very early, during the acute phase of the ACS, when patients may still be in shock, processing what has happened and focusing on their physical recovery. Thus, patients may not have been in the best position to benefit from such an early PI, as the cognitive and motivational resources required were not optimal.

In conclusion, CBT and PPT-based PIs in patients with ACS may have additional benefits compared to those produced by conventional CR programs, particularly at a psychological level. Further larger trials to prove these preliminary findings are warranted.

## Data Availability

The raw data supporting the conclusions of this article will be made available by the authors upon request.

## References

[ref1] Abreu A. Pesah E. Supervia M. Turk-Adawi K. Bjarnason-Wehrens B. Lopez-Jimenez F.. (2019). Cardiac rehabilitation availability and delivery in Europe: how does it differ by region and compare with other high-income countries? Eur. J. Prev. Cardiol. 26, 1131–1146. doi: 10.1177/2047487319827453 30782007

[ref2] Anderson L. Oldridge N. Thompson D. R. Zwisler A.-D. Rees K. Martin N.. (2016). Exercise-based cardiac rehabilitation for coronary heart disease: Cochrane systematic review and Meta-analysis. J. Am. Coll. Cardiol. 67, 1–12. doi: 10.1016/j.jacc.2015.10.044 26764059

[ref3] Arnold S. V. Smolderen K. G. Buchanan D. M. Li Y. Spertus J. A. (2012). Perceived stress in myocardial infarction. J. Am. Coll. Cardiol. 60, 1756–1763. doi: 10.1016/j.jacc.2012.06.044 23040574 PMC3601381

[ref4] Baayen R. H. Davidson D. J. Bates D. M. (2008). Mixed-effects modeling with crossed random effects for subjects and items. J. Mem. Lang. 59, 390–412. doi: 10.1016/j.jml.2007.12.005

[ref5] Boehm J. K. (2021). Positive psychological well-being and cardiovascular disease: exploring mechanistic and developmental pathways. Soc. Personal. Psychol. Compass 15:12599. doi: 10.1111/spc3.12599 PMC9285725 35860033

[ref6] Boehm J. K. Kubzansky L. D. (2012). The heart’s content: the association between positive psychological wellbeing and cardiovascular health. Psychol. Bull. 138, 655–691. doi: 10.1037/a0027448 22506752

[ref7] Bolier L. Haverman M. Westerhof G. J. Riper H. Smit F. Bohlmeijer E. (2013). Positive psychology interventions: a meta-analysis of randomized controlled studies. BMC Public Health 13:119. doi: 10.1186/1471-2458-13-119 23390882 PMC3599475

[ref8] Carney R. M. Freedland K. E. (2017). Depression and coronary heart disease. Nat. Rev. Cardiol. 14, 145–155. doi: 10.1038/nrcardio.2016.181 27853162

[ref9] Carver C. S. (1997). You want to measure coping but your protocol’ too long: consider the brief cope. Int. J. Behav. Med. 4, 92–100. doi: 10.1207/s15327558ijbm0401_6 16250744

[ref10] Carver C. S. (2019). “Coping” in The Cambridge handbook of psychology, health and medicine. eds. Llewellyn C. D. Ayers S. McManus C. Newman S. Petrie K. J. Revenson T. A. (Cambridge: Cambridge University Press), 114–118.

[ref11] Chida Y. Hamer M. (2008). Chronic psychosocial factors and acute physiological responses to laboratory-induced stress in healthy populations: a quantitative review of 30 years of investigations. Psychol. Bull. 134, 829–885. doi: 10.1037/a0013342 18954159

[ref12] Chida Y. Steptoe A. (2009). The Association of Anger and Hostility with Future Coronary Heart Disease. A Meta-analytic review of prospective evidence. J. Am. Coll. Cardiol. 53, 936–946. doi: 10.1016/j.jacc.2008.11.044 19281923

[ref13] Dickens C. Cherrington A. Adeyemi I. Roughley K. Bower P. Garrett C.. (2013). Characteristics of psychological interventions that improve depression in people with coronary heart disease: a systematic review and meta-regression. Psychosom. Med. 75, 211–221. doi: 10.1097/PSY.0b013e31827ac009 23324874

[ref14] DuBois C. M. Lopez O. V. Beale E. E. Healy B. C. Boehm J. K. Huffman J. C. (2015). Relationships between positive psychological constructs and health outcomes in patients with cardiovascular disease: a systematic review. Int. J. Cardiol. 195, 265–280. doi: 10.1016/j.ijcard.2015.05.121 26048390 PMC4487518

[ref15] Fihn S. D. Blankenship J. C. Alexander K. P. Bittl J. A. Byrne J. G. Fletcher B. J.. (2014). 2014 ACC/AHA/AATS/PCNA/SCAI/STS focused update of the guideline for the diagnosis and Management of Patients with Stable Ischemic Heart Disease. J. Am. Coll. Cardiol. 64, 1929–1949. doi: 10.1016/j.jacc.2014.07.017 25077860

[ref16] Gratz K. L. Roemer L. (2004). Multidimensional assessment of emotion regulation and dysregulation: development, factor structure, and initial validation of the difficulties in emotion regulation scale. J. Psychopathol. Behav. Assess. 26, 41–54. doi: 10.1023/B:JOBA.0000007455.08539.94

[ref17] Hamer M. Malan L. (2010). Psychophysiological risk markers of cardiovascular disease. Neurosci. Biobehav. Rev. 35, 76–83. doi: 10.1016/j.neubiorev.2009.11.004 19909773

[ref18] Hervás G. Jódar R. (2008). Adaptación al castellano de la Escala de Dificultades en la Regulación Emocional. Clin. Salud. 19, 139–156.

[ref19] Huffman J. C. Millstein R. A. Mastromauro C. A. Moore S. V. Celano C. M. Bedoya C. A.. (2016). A positive psychology intervention for patients with an acute coronary syndrome: treatment development and proof-of-concept trial. J. Happiness Stud. 17, 1985–2006. doi: 10.1007/s10902-015-9681-1 28082831 PMC5222616

[ref20] Knuuti J. Wijns W. Saraste A. Capodanno D. Barbato E. Funck-Brentano C.. (2020). 2019 ESC guidelines for the diagnosis and management of chronic coronary syndromes. Eur. Heart J. 41, 407–477. doi: 10.1093/eurheartj/ehz425 31504439

[ref21] Kubzansky L. D. Huffman J. C. Boehm J. K. Hernandez R. Kim E. S. Koga H. K.. (2018). Positive psychological wellbeing and cardiovascular disease. J. Am. Coll. Cardiol. 72, 1382–1396. doi: 10.1016/j.jacc.2018.07.042 30213332 PMC6289282

[ref22] Labarthe D. R. Kubzansky L. D. Boehm J. K. Lloyd-Jones D. M. Berry J. D. Seligman M. E. P. (2016). Positive cardiovascular health. J. Am. Coll. Cardiol. 68, 860–867. doi: 10.1016/j.jacc.2016.03.608 27539179

[ref23] Lakens D. (2022). Sample size justification. Collabra Psychol 8:33267. doi: 10.1525/collabra.33267

[ref24] Lazarus R. S. Folkman S. (1984). Stress, appraisal, and coping. New York: Springer publishing company.

[ref25] Levine G. N. Cohen B. E. Commodore-Mensah Y. Fleury J. Huffman J. C. Khalid U.. (2021). Psychological health, wellbeing, and the mind-heart-body connection a scientific statement from the American Heart Association. Circulation 143, E763–E783. doi: 10.1161/CIR.0000000000000947 33486973

[ref26] Linden W. (2000). Psychological treatments in cardiac rehabilitation. J. Psychosom. Res. 48, 443–454. doi: 10.1016/S0022-3999(99)00094-X 10880665

[ref27] Linden W. (2013). How many Meta-analyses does it take to settle a question? Psychosom. Med. 75, 332–334. doi: 10.1097/PSY.0b013e318295e046 23630308

[ref28] Linden W. Phillips M. J. Leclerc J. (2007). Psychological treatment of cardiac patients: a meta-analysis. Eur. Heart J. 28, 2972–2984. doi: 10.1093/eurheartj/ehm504 17984133

[ref29] Lovallo W. R. Gerin W. (2003). Psychophysiological reactivity: mechanisms and pathways to cardiovascular disease. Psychosom. Med. 65, 36–45. doi: 10.1097/01.PSY.0000033128.44101.C1 12554814

[ref30] Magán I. Casado L. Jurado-Barba R. Barnum H. Redondo M. M. Hernandez A. V.. (2021). Efficacy of psychological interventions on psychological outcomes in coronary artery disease: systematic review and meta-analysis. Psychol. Med. 51, 1846–1860. doi: 10.1017/S0033291720000598 32249725

[ref31] Magán I. Jurado-Barba R. Casado L. Barnum H. Jeon A. Hernandez A. V.. (2022). Efficacy of psychological interventions on clinical outcomes of coronary artery disease: systematic review and meta-analysis. J. Psychosom. Res. 153:110710. doi: 10.1016/j.jpsychores.2021.110710 34999380

[ref43] McDonagh T. A. Metra M. Adamo M. Gardner R. S. Baumbach A. Böhm M.. (2021). ESC Guidelines for the diagnosis and treatment of acute and chronic heart failure. Eur Heart J. 42, 3599–3726. doi: 10.1093/eurheartj/ehab368 34447992

[ref32] Michalsen A. Grossman P. Lehmann N. Knoblauch N. T. Paul A. Moebus S.. (2005). Psychological and quality-of-life outcomes from a comprehensive stress reduction and lifestyle program in patients with coronary artery disease: results of a randomized trial. Psychother. Psychosom. 74, 344–352. doi: 10.1159/000087781 16244510

[ref33] Moghei M. Oh P. Chessex C. Grace S. L. (2019). Cardiac rehabilitation quality improvement. J. Cardiopulm. Rehabil. Prev. 39, 226–234. doi: 10.1097/HCR.0000000000000396 30720641

[ref34] Mohammadi N. Aghayousefi A. Nikrahan G. R. Adams C. N. Alipour A. Sadeghi M.. (2018). A randomized trial of an optimism training intervention in patients with heart disease. Gen. Hosp. Psychiatry 51, 46–53. doi: 10.1016/j.genhosppsych.2017.12.004 29316450

[ref35] Montgomery P. Grant S. Mayo-Wilson E. Macdonald G. Michie S. Hopewell S.. (2018). Reporting randomised trials of social and psychological interventions: the CONSORT-SPI 2018 extension. Trials 19:407. doi: 10.1186/s13063-018-2733-1 30060754 PMC6066921

[ref36] Morán C. Landero R. González M. T. (2010). COPE-28: un análisis psicométrico de la versión en español del Brief COPE. Univ. Psychol. 9, 543–552.

[ref37] Nicholson A. Kuper H. Hemingway H. (2006). Depression as an aetiologic and prognostic factor in coronary heart disease: a meta-analysis of 6362 events among 146 538 participants in 54 observational studies. Eur. Heart J. 27, 2763–2774. doi: 10.1093/eurheartj/ehl338 17082208

[ref38] Nikrahan G. R. Laferton J. A. C. Asgari K. Kalantari M. Abedi M. R. Etesampour A.. (2016). Effects of positive psychology interventions on risk biomarkers in coronary patients: a randomized, wait-list controlled pilot trial. Psychosomatics 57, 359–368. doi: 10.1016/j.psym.2016.02.007 27129358 PMC4902729

[ref39] Oldridge N. Höfer S. Mcgee H. Saner H. (2022). Evaluation of health-related quality of life in cardiovascular research: a call for action. Eur. J. Prev. Cardiol. 29, E79–E81. doi: 10.1093/eurjpc/zwab008 34038521

[ref40] Ossola P. Gerra M. L. De Panfilis C. Tonna M. Marchesi C. (2018). Anxiety, depression, and cardiac outcomes after a first diagnosis of acute coronary syndrome. Health Psychol. 37, 1115–1122. doi: 10.1037/hea0000658 30307271

[ref41] Otero J. M. Luengo A. Romero F. Gómez J. A. Castro C. (1998). Psicología de la Personalidad. Manual de Prácticas. Barcelona: Ariel Practicum.

[ref42] Poffley A. Thomas E. Grace S. L. Neubeck L. Gallagher R. Niebauer J.. (2017). A systematic review of cardiac rehabilitation registries. Eur. J. Prev. Cardiol. 24, 1596–1609. doi: 10.1177/2047487317724576 28762761

[ref44] Richards S. H. Anderson L. Jenkinson C. E. Whalley B. Rees K. Davies P.. (2018). Psychological interventions for coronary heart disease: Cochrane systematic review and meta-analysis. Eur. J. Prev. Cardiol. 25, 247–259. doi: 10.1177/2047487317739978 29212370

[ref45] Roest A. M. Martens E. J. de Jonge P. Denollet J. (2010). Anxiety and risk of incident coronary heart disease. J. Am. Coll. Cardiol. 56, 38–46. doi: 10.1016/j.jacc.2010.03.034 20620715

[ref46] Rozanski A. (2014). Behavioral cardiology. J. Am. Coll. Cardiol. 64, 100–110. doi: 10.1016/j.jacc.2014.03.047 24998134

[ref47] Rozanski A. Bavishi C. Kubzansky L. D. Cohen R. (2019). Association of Optimism with cardiovascular events and all-cause mortality: a systematic review and Meta-analysis. JAMA Netw. Open 2:12200. doi: 10.1001/jamanetworkopen.2019.12200 PMC6777240 31560385

[ref48] Russ T. C. Stamatakis E. Hamer M. Starr J. M. Kivimaki M. Batty G. D. (2012). Association between psychological distress and mortality: individual participant pooled analysis of 10 prospective cohort studies. Brit. Med. J. 345:e4933. doi: 10.1136/bmj.e4933 22849956 PMC3409083

[ref49] Rutledge T. Redwine L. S. Linke S. E. Mills P. J. (2013). A Meta-analysis of mental health treatments and cardiac rehabilitation for improving clinical outcomes and depression among patients with coronary heart disease. Psychosom. Med. 75, 335–349. doi: 10.1097/PSY.0b013e318291d798 23630306

[ref50] Sanjuan P. Montalbetti T. Pérez-García A. M. Bermúdez J. Arranz H. Castro A. (2016). A randomised trial of a positive intervention to promote wellbeing in cardiac patients. Appl. Psychol. Health Well Being 8, 64–84. doi: 10.1111/aphw.12062 26876425

[ref51] Scheier M. F. Carver C. S. Bridges M. W. (1994). Distinguishing optimism from neuroticism (and trait anxiety, self-mastery, and self-esteem): a reevaluation of the life orientation test. J. Pers. Soc. Psychol. 67, 1063–1078. doi: 10.1037/0022-3514.67.6.1063 7815302

[ref52] Schulz K. F. Altman D. G. Moher D. (2010). CONSORT 2010 statement: updated guidelines for reporting parallel group randomised trials. BMC Med. 8:18. doi: 10.1186/1741-7015-8-18 20334633 PMC2860339

[ref53] Schwartz A. R. Gerin W. Davidson K. W. Pickering T. G. Brosschot J. F. Thayer J. F.. (2003). Toward a causal model of cardiovascular responses to stress and the development of cardiovascular disease. Psychosom. Med. 65, 22–35. doi: 10.1097/01.PSY.0000046075.79922.61 12554813

[ref54] Seligman M. (2011). Flourish. New York: Free Press.

[ref55] Seligman M. Steen T. Park N. Peterson C. (2005). Positive psychology progress: empirical validation of interventions. Am. Psychol. 60, 410–421. doi: 10.1037/0003-066X.60.5.410 16045394

[ref56] Spielberger C. D. (1999). Sate-trait anger expression Inventory-2: STAXI-2. Lutz, Florida: PAR.

[ref57] Spielberger C. D. Miguel Tobal J. J. Casado M. I. Cano Vindel A. (2001). Inventario de Expresión de Ira Estado Rasgo 2–STAXI 2. Madrid: TEA.

[ref58] Stenvall H. Tierala I. Räsänen P. Laine M. Sintonen H. Roine R. P. (2017). Long-term clinical outcomes, health-related quality of life, and costs in different treatment modalities of stable coronary artery disease. Eur. Heart J. Qual. Care Clin. Outcomes 3, 74–82. doi: 10.1093/ehjqcco/qcw024 28927186

[ref59] Steptoe A. (2019). Happiness and health. Annu. Rev. Public Health 40, 339–359. doi: 10.1146/annurev-publhealth-040218-044150 30601719

[ref60] Steptoe A. Kivimäki M. (2013). Stress and cardiovascular disease: an update on current knowledge. Annu. Rev. Public Health 34, 337–354. doi: 10.1146/annurev-publhealth-031912-114452 23297662

[ref61] Steptoe A. Wardle J. Marmot M. (2005). Positive affect and health-related neuroendocrine, cardiovascular, and inflammatory processes. Proc. Natl. Acad. Sci. USA. 102: 6508–6512. doi: 10.1073/pnas.0409174102 PMC1088362 15840727

[ref62] Supervia M. Turk-Adawi K. Lopez-Jimenez F. Pesah E. Ding R. Britto R. R.. (2019). Nature of cardiac rehabilitation around the globe. EClinicalMedicine 13, 46–56. doi: 10.1016/j.eclinm.2019.06.006 31517262 PMC6733999

[ref63] Tejero A. Guimerá E. Farré J. Peri J. (1986). Uso clínico del HAD (Hospital Anxiety and Depression Scale) en población psiquiátrica: Un estudio de su sensibilidad, fiabilidad y validez. Rev. Depart. Psiquiatría Fac. Med. Barcelona 13, 233–238.

[ref64] Tönis K. J. M. Kraiss J. T. Linssen G. C. M. Bohlmeijer E. T. (2023). The effects of positive psychology interventions on wellbeing and distress in patients with cardiovascular diseases: a systematic review and Meta-analysis. J. Psychosom. Res. 170:111328. doi: 10.1016/j.jpsychores.2023.111328 37098284

[ref65] Tully P. J. Winefield H. R. Baker R. A. Denollet J. Pedersen S. S. Wittert G. A.. (2015). Depression, anxiety and major adverse cardiovascular and cerebrovascular events in patients following coronary artery bypass graft surgery: a five year longitudinal cohort study. Biopsychosoc. Med. 9:14. doi: 10.1186/s13030-015-0041-5 26019721 PMC4445298

[ref66] Vilagut G. María Valderas J. Ferrer M. Garin O. López-García E. Alonso J. (2008). Interpretación de los cuestionarios de salud SF-36 y SF-12 en España: componentes físico y mental. Med. Clin. 130, 726–735. doi: 10.1157/13121076 18570798

[ref67] Vos J. (2021). Cardiovascular disease and meaning in life: a systematic literature review and conceptual model. Palliat. Support. Care 19, 367–376. doi: 10.1017/S1478951520001261 33960285

[ref68] Ware J. E. Kosinski M. Keller S. D. (1996). A 12-item short-form health survey. Med. Care 34, 220–233. doi: 10.1097/00005650-199603000-00003 8628042

[ref69] Wirtz P. H. von Känel R. (2017). Psychological stress, inflammation, and coronary heart disease. Curr. Cardiol. Rep. 19:111. doi: 10.1007/s11886-017-0919-x 28932967

[ref70] Zigmond A. S. Snaith R. P. (1983). The hospital anxiety and depression scale. Acta Psychiatr. Scand. 67:716. doi: 10.1111/j.1600-0447.1983.tb09716.x 6880820

