# Supplementary material for: PsicoCare: a pilot randomized controlled trial testing a psychological intervention combining cognitive-behavioral treatment and positive psychology therapy in acute coronary syndrome patients
Source: Front Psychol. 2024 Nov 19;15:1420137. doi: 10.3389/fpsyg.2024.1420137 (PMC11611559; doi:10.3389/fpsyg.2024.1420137)
Supplement: Supplementary file 1 [file Table_1.DOCX]

**CONSORT-SPI 2018 Checklist**

| **SECTION** | **ITEM #** | **CONSORT 2010** | **CONSORT-SPI**  **2018** | **REPORTED ON PAGE #** |
| --- | --- | --- | --- | --- |
| **TITLE AND ABSTRACT** | | | | |
|  | 1a | Identification as a randomised trial in the title^§^ |  | Pg 1 |
|  | 1b | Structured summary of trial design, methods, results, and conclusions (for specific guidance see CONSORT for Abstracts)^§^ | Refer to CONSORT extension for social and psychological intervention trial abstracts | Pg 2 |
| **INTRODUCTION** | | | | |
| Background and  Objectives | 2a | Scientific background and explanation of rationale ^§^ |  | Pg 3 |
|  | 2b | Specific objectives or hypotheses ^§^ | If pre-specified, how the intervention was hypothesied to work | Pg 3, 76-81 lines |
| **METHODS** | | | | |
| Trial Design | 3a | Describe of trial design (such as parallel, factorial), including allocation ratio ^§^ | If the unit of random assignment is not the individual, please refer to CONSORT for Cluster Randomized Trials | Pg 3, 83-87 lines |
|  | 3b | Important changes to methods after trial commencement (such as eligibility criteria), with reasons |  | There was not any change after trial commencement |
| Participants | 4a | Eligibility criteria for participants^§^ | When applicable, eligibility criteria for settings and those delivering the interventions | Pg 3-4, 90-95 lines |
|  | 4b | Settings and locations where the data were collected |  | Pg 3-4, 89-104 lines |
| Interventions | 5 | The interventions for each group with sufficient details to allow replication, including how and when they are actually administered ^§^ |  | Pg 4-5, 131-155 lines and Supplementary figure 1 and Supplementary Table 1 |
|  | 5a |  | Extent to which interventions were actually delivered by providers and taken up by participants as planned | Flow chart diagram, Figure 1 |
|  | 5b |  | Where other informational materials about delivering the intervention can be accessed | Supplementary figure 1 and Supplementary Table 1 |
|  | 5c |  | When applicable, how intervention providers were assigned to each group | Not applicable |
| Outcomes | 6a | Completely defined pre-specified outcomes, including how and when they were assessed^§^ |  | Pg 4, 115-120 lines.  Pg 5, 157-183 lines  Supplementary Figure 1 |
|  | 6b | Any changes to trial outcomes after the trial commenced, with reasons |  | No changes were made |
| Sample Size | 7a | How sample size was determined^§^ |  | As participants recruitment was incidental, a sample size estimation was not conducted. |
|  | 7b | When applicable, explanation of any interim analyses and stopping guidelines |  | Not applicable |
| **RANDOMISATION** | | | | |
| Sequence  generation | 8a | Method used to generate the random allocation sequence |  | Pg 4, 99-104 lines |
|  | 8b | Type of randomisation; detail of any restriction (such as blocking and block size)^§^ |  | Pg 4, 99-104 lines |
| Allocation concealment mechanism | 9 | Mechanism used to implement the random allocation sequence, describing any steps taken to conceal the sequence until interventions were assigned^§^ |  | Pg 4, 99-104 lines |
| Implementation | 10 | Who generated the random allocation sequence, who enrolled participants, and who assigned participants to interventions^§^ |  | Pg 4, 99-104 lines |
| Awareness of assignment | 11a | Who was aware of intervention assignment after allocation (for example, participants, providers, those assessing outcomes), and how any masking was done |  | Pg 4, 99-104 lines |
|  | 11b | If relevant, description of the similarity of interventions |  | Pg 5, 131-155 lines and Supplementary Figure 1 and Table 1 |
| Analytical  methods | 12a | Statistical methods used to compare group outcomes^§^ | How missing data were handled, with details of any imputation method | Pg 6, 185-209 lines |
|  | 12b | Methods for additional analyses, such as subgroup analyses, adjusted analyses, and process evaluations |  | Pg 6, 185-209 lines |
| **RESULTS** | | | | |
| Participant flow (a diagram is strongly recommended) | 13a | For each group, the numbers randomly assigned, receiving the intended intervention, and analysed for the outcomes^§^ | Where possible, the number approached, screened, and eligible prior to random assignment, with reasons for non-enrolment | Flow chart, Figure 1 |
|  | 13b | For each group, losses and exclusions after randomisation, together with reasons^§^ |  | Flow chart, Figure 1 |
| Recruitment | 14a | Dates defining the periods of recruitment and follow-up |  | Pg. 3, 84-85 lines |
|  | 14b | Why the trial ended or was stopped |  | Recruitment was stopped in order to analyze the viability of the intervention as well as to check the coordination between the different hospital departments. It has not been specified in the manuscript because it is not determinant for the trial results and conclusions. |
| Baseline data | 15 | A table showing baseline characteristics for each group^§^ | Include socioeconomic variables where applicable | Pg. 7-8, 214-220 lines.  Supplementary table 6 |
| Numbers analysed | 16 | For each group, number included in each analysis and whether the analysis was by original assigned groups^§^ |  | Pg. 7, 212-214 lines and Flow chart diagram |
| Outcomes and estimation | 17a | For each outcome, results for each group, and the estimated effect size and its precision (such as 95% confidence interval)^§^ | Indicate availability of trial data | Pg. 7 and 8, 227-271lines.  Tables 1, 2 and 3  Supplementary Tables 2, 3, 4, 5, and 7 |
|  | 17b | For binary outcomes, the presentation of both absolute and relative effect sizes is recommended |  | Pg. 8, 264-271 lines  Table 3, and Supplementary Table 4 |
| Ancillary analyses | 18 | Results of any other analyses performed, including subgroup analyses, adjusted analyses, and process evaluations, distinguishing pre-specified from exploratory |  | There were not any ancillary analyses. |
| Harms | 19 | All important harms or unintended effects in each group (for specific guidance see CONSORT for Harms) |  | There were not any important harms or unintended effects. |
| **DISCUSSION** | | | | |
| Limitations | 20 | Summarize the main results (including an overview of concepts, themes, and types of evidence available), link to the review questions and objectives, and consider the relevance to key groups. | Trial limitations, addressing sources of potential bias, imprecision, and, if relevant, multiplicity of analyses | Summarize the main results: pg. 8. 283-284 lines.  Limitations are discussed along all de Discussion section. |
| Generalisability | 21 | Discuss the limitations of the scoping review process. | Generalisability (external validity, applicability) of the trial findings^§^ | Not applicable. External validity has not been assessed. Intervention applicability is discussed along the Discussion section. |
| Interpretation | 22 | Provide a general interpretation of the results with respect to the review questions and objectives, as well as potential implications and/or next steps. | Interpretation consistent with results, balancing benefits and harms, and considering other relevant evidence | Pg. 8-10, 285-385 lines. |
| **IMPORTANT INFORMATION** | | | | |
| Registration | 23 | Registration number and name of trial registry |  | Pg. 3, line 85.  (Registered at Clinical Trials, NCT05287061. Unique protocol ID: 17/171). |
| Protocol | 24 | Where the full trial protocol can be accessed, if available |  | https://clinicaltrials.gov/study/NCT05287061 |
| Declaration of Interests | 25 | Sources of funding and other support; role of funders | Declaration of any other potential interests | Funding: pg. 11, 411-412.  Conflict of interest: pg. 11, 391-399 lines. |
| Stakeholder investments | 26a |  | Any involvement of the intervention developer in the design, conduct, analysis, or reporting of the trial | There were not any stakeholder investments. |
|  | 26b |  | Other stakeholder involvement in trial design, conduct, or analyses | There were not any other stakeholder involvements. |
|  | 26c |  | Incentives offered as part of the trial | There were not any incentives offered. |

This table lists items from the CONSORT 2010 checklist (with some modifications for social and psychological intervention trials) and additional items in the CONSORT-SPI 2018 extension. Empty rows in the ‘CONSORT-SPI 2018’ column indicate that there is no extension to the CONSORT 2010 item

*We strongly recommended that the CONSORT-SPI 2018 Explanation and Elaboration (E&E) document be reviewed when using the CONSORT-SPI 2018 checklist for important clarifications on each item

§An extension item for cluster trials exists for this CONSORT 2010 item

This checklist is derived from:

- Montgomery, P., Grant, S., Mayo-Wilson, E., Macdonald, G., Michie, S., Hopewell, S., & Moher, D. (2018). Reporting randomised trials of social and psychological interventions: the CONSORT-SPI 2018 Extension. *Trials*, *19*(1), 407.
- Grant, S., Mayo-Wilson, E., Montgomery, P., Macdonald, G., Michie, S., Hopewell, S., & Moher, D. (2018). CONSORT-SPI 2018 Explanation and Elaboration: guidance for reporting social and psychological intervention trials. *Trials*, *19*(1), 406.
- Schulz, K. F., Altman, D. G., & Moher, D. (2010). CONSORT 2010 Statement: updated guidelines for reporting parallel group randomised trials. *BMJ*, *340*, c332.

Montgomery 2018 and Grant 2018 were distributed under the terms of the Creative Commons Attribution 4.0 International License ([http://creativecommons.org/licenses/by/4.0/](https://urldefense.com/v3/__http:/creativecommons.org/licenses/by/4.0/__;!!C5qS4YX3!CdcaroZO5hxp8FYacDXbUMpysBqFhPEDSGHr-PBP-6JeLAF0ufQ5lz_kopWiil164numaGA4JU3gaxY-WqOg1G1v39F-mLM$)). Schulz 2010 was distributed under the terms of a Creative Commons Attribution Non-commercial License (<https://creativecommons.org/licenses/by-nc/2.0/>). We have revised the checklists as published to include an extra column for “reporting on page #”.
