# Supplementary material for: PsicoCare: a pilot randomized controlled trial testing a psychological intervention combining cognitive-behavioral treatment and positive psychology therapy in acute coronary syndrome patients
Source: Front Psychol. 2024 Nov 19;15:1420137. doi: 10.3389/fpsyg.2024.1420137 (PMC11611559; doi:10.3389/fpsyg.2024.1420137)
Supplement: Supplementary file 2 [file Data_Sheet_1.docx]

**Supplemental material:**

PSICOCARE: A pilot randomized controlled trial testing a psychological intervention combining cognitive-behavioral treatment and positive psychology therapy in acute coronary syndrome patients

**Brief title:** *PsicoCare: A psychological intervention RCT in ACS patients*

**Inés Magán, PhD^1, 2*^** **^†^ ; Rosa Jurado-Barba, PhD^1,2^** **^†^; Guillermo Moreno, RN, PhD ^3, 4, 5^; María Paz Ayán-Sanz, MD, PhD^6^; Juan Izquierdo-Garcia, PT^6^; Guido Corradi, PhD^1, 10^; Rocio Tello, MD, PhD ^3,^ 4; and Héctor Bueno, MD, PhDb, ^3, 4, 7, 8, 9^** **^†^**

^1^Facultad HM de Ciencias de la Salud de la Universidad Camilo José Cela - Villanueva de la Cañada 28692, Madrid, Spain

^2^Instituto de Investigación Sanitaria HM Hospitales, Madrid, Spain

^3^Instituto de Investigación Hospital 12 de Octubre (imas12), Madrid, Spain

^4^Department of Cardiology, Hospital Universitario 12 de Octubre, Madrid, Spain

^5^Facultad de Enfermería, Fisioterapia y Podología, Universidad Complutense de Madrid, Madrid, Spain

^6^Department of Rehabilitation, Hospital Universitario 12 de Octubre, Madrid, Spain

^7^Multidisciplinary Translational Cardiovascular Research Group, Centro Nacional de Investigaciones Cardiovasculares (CNIC), Madrid, Spain.

^8^Facultad de Medicina, Universidad Complutense de Madrid, Madrid, Spain

^9^Centro de Investigación Biomédica en Red Enfermedades Cardiovaculares (CIBERCV), Madrid, Spain.

^10^Departamento de Psicología, Universidad Villanueva

*** Correspondence:**Inés Magán
imagan@ucjc.edu

**†**These authors contributed equally to this work and share senior authorship

**October 14^th^, 2024**

**Content of Supplemental Material:**

- **Supplemental Material 1:** *PsicoCare* trial protocol description and *PsicoCare* intervention program description.
  - Supplementary Figure 1: *PsicoCare* Trial Study Protocol
  - Supplementary Table 1: *PsicoCare* intervention program description
- **Supplemental Material 2:** software and functions used for main analysis.
- **Supplemental Material 3:** tables for comparative fit indexes and coefficients for all models.
  - Supplementary Table 2: *Health Pills:* Prediction models coefficients and inferential statistics for psychological outcomes.
  - Supplementary Table 3: *PsicoCare*: Prediction models coefficients and inferential statistics for psychological outcomes.
  - Supplementary Table 4: *PsicoCare*: Prediction models coefficients and inferential statistics for ergometry outcomes.
  - Supplementary Table 5: *PsicoCare:* Prediction models coefficients and inferential statistics for biochemical and anthropometrical outcomes.
- **Supplemental Material 4:** *Health Pills efficacy data.*
  - Supplementary Table 6. *Health Pills*: Psychological outcomes descriptive statistics and change score between time 1 and time 2 assessment

**Supplemental Material 1:** *PsicoCare* trial protocol description (online Figure 1) and *PsicoCare* intervention program description (online Table 1).

**Supplementary Figure 1. PsicoCare Trial Study Protocol**


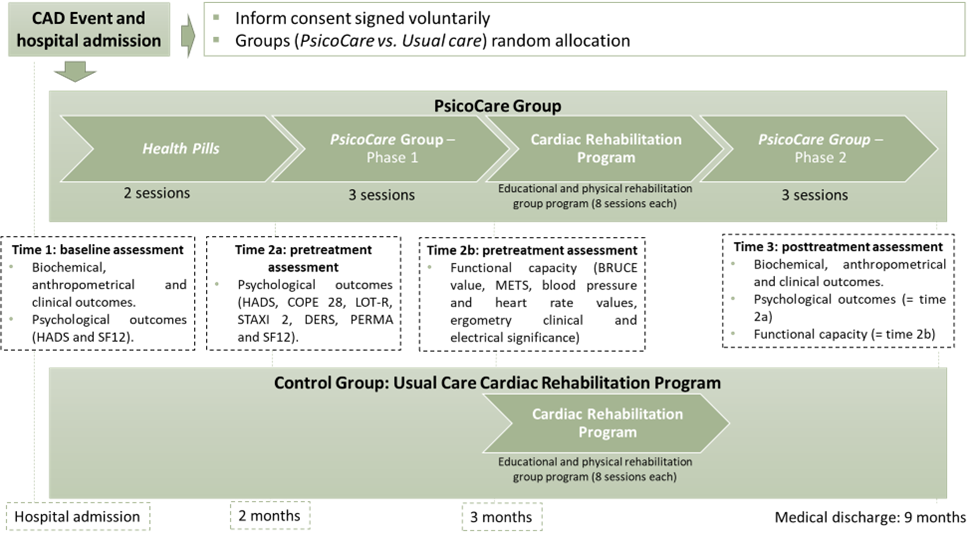


**Supplementary Table 1.** *PsicoCare intervention program description*

| **Treatment phase** | **Treatment characteristics** | **Description** | | |
| --- | --- | --- | --- | --- |
|  |  | **Session** | **Objetives** | **Techniques** |
| ***Health pills*** | - Two individual 1-hour weekly sessions. - Provider: health psychologist. | 1. *Health pills* I | 1. Express and discharge emotional distress. 2. Develop CVD learning. 3. Enhance personal growth after the event and change commitment. 4. Develop emotional and distress coping skills. | - Emotional discharging. - Psychoeducation about CVD and emotions. - Motivational interview. - Relaxation (diaphragmatic breathing) training. - Self-instructions training. - Attention focus on daily positive things or experiences training. |
|  |  | 2. *Health pills* II | 1. Review intersessions period. 2. Strength emotional and skills coping trained. 3. Start to improve depressive mood. | - Patient interview. - Review and strengthen coping skills worked on session one (relaxation, self-instructions, and attention focus on positive things). - Behavioral activation + attention focus on daily positive things or experiences training. |
| ***PsicoCare*** | - Six group 2-hour weekly sessions divided into two phases -phase 1, included the first three sessions and phase 2, included the other three, developed after usual care program-. - Provider: two health psychologists. | 1. Personal growth I | 1. Develop therapeutic alliance and group relationships. 2. Review general patient state. 3. Enhance personal growth through. 4. Consolidate emotional distress coping skills. 5. Homework | - Open and motivational interview and group exercise. - Motivational interview: 5-years life plan based on their own purposes. - Skills developed on Pills Health stage (relaxation, self-instructions, and behavioral activation + attention focus on positive things) review and consolidation. - Homework: put into practice the skills worked and developed their own life-plan. |
|  |  | 2. Emotions I | 1. Review homework and weekly period. 2. Develop emotional knowledge: negative emotions. 3. Develop and consolidate negative emotions identification and skills coping to manage them. 4. Introduction to ABC emotion model and cognitive restructuring. 5. Review and consolidate their own purposes. 6. Homework. | - Homework and weekly review. - Negative emotions psychoeducation and emotion identification skills training. - Training on using the skills developed on Pills Health (relaxation training, self-instructions, behavioral activation + positive attention focus training) stage for managing negative emotions. - Cognitive restructuring. - Homework: negative emotion manage self-record and life purposes planning daily implementing. |
|  |  | 3. Emotions II | 1. Review homework and weekly period. 2. Develop emotional knowledge: positive emotions. 3. Cultivate and enhance personal positive emotions. 4. Consolidate skills to manage negative emotions. 5. Review and consolidate own purposes. 6. Homework. | - Homework and weekly review. - Positive emotions psychoeducation. - Seligman exercises to identify and develop personal strengths. - Relaxation training, self-instructions, behavioral activation + positive attention focus training, and cognitive restructuring for managing negative emotions. - Homework: negative emotion manage self-record, Seligman exercises and life purposes planning daily implementing. |
|  |  | *Cardiac usual care rehabilitation program: educational program + physical rehabilitation training* | | |
|  |  | 1. Personal proposals consolidation I | 1. Review intersession period and general patient´s state. 2. Renewal personal proposals and enhance personal change motives commitment for their new healthy lifestyle. 3. Enhance an active personal role and a personal skill plan to manage difficult situations. 4. Homework. | - Homework and intersession period review. - Motivational interviewing. - Problem-solution and decision-making skill training. - Negative emotions skills and Seligman personal strengths exercises review for promoting, respectively, negative and positive emotions. - Homework. |
|  |  | 1. Personal proposals consolidation II | 1. Review weekly period and general patient´s state. 2. Enhance the new personal proposals and personal change motives commitment for their new healthy lifestyle. 3. Enhance an active personal role and a personal skill plan to manage difficult situations. 4. Homework. | - Homework and intersession period review. - Motivational interviewing. - Problem-solution and decision-making skill training. - Negative emotions skills and Seligman personal strengths exercises review for promoting, respectively, negative and positive emotions. - Homework. |
|  |  | 1. Personal growth consolidation II and relapse prevention | 1. Review weekly period and general patient´s state. 2. Review what they have learned and how they and their lives have changed. 3. Develop a positive orientation to relapse events. 4. Develop a personal plan to cope with future relapses. 5. Group farewell ending treatment program. | - Homework and intersession period review. - Motivational interviewing. - Relapse prevention training program: psychoeducation, learning the difference between an isolate lapse event and a relapse, reviewing personal changes and which skills are really useful for them, anticipate future personal risk episodes, and develop and imagination training a personal coping plan. |

**Supplemental Material 2: Software and functions used for main analysis.**

The *parameters* function of the ‘easystats’ package was used (Lüdecke et al., 2021), with Satterthwaite method to produce the inferential statistics, confidence intervals and *p*-values. All models' main assumptions (linearity, normality residuals, and homoscedasticity) were visually checked following *check_model* function from ‘easystats’ package (Lüdecke et al., 2021). Also, *emmeans* function from the ‘emmeans’ package (Lenth, 2015) was used to create the predicted marginal means, contrasts, and comparisons for fixed effects of models.

**Supplemental Material 3: Tables for comparative fit indexes and coefficients for all models**

**Supplementary *Table 2.*** *Health Pills: Prediction models coefficients and inferential statistics for psychological outcomes*

|  | **M1** |  |  |  |  | **M2** |  |  |  |  |  |
| --- | --- | --- | --- | --- | --- | --- | --- | --- | --- | --- | --- |
| **Predicted value** | **b 95% CI**  ***β* p-value** | **AICc** | **R2**  **Conditional** | **R2**  **Marginal** | **ICC** | **b_treatment_** **95% CI**  ***β* p-value** | **biam**  **95% CI**  ***β* p-value** | **AICc** | **R2**  **Conditional** | **R2**  **Marginal** | **ICC** |
| **Anxiety (HADS-A)** | -0.13 [-1.75, 1.50]  0.04 0.855 | 781.03 | 0.50 | 0.07 | 0.54 | 0.17 [-1.29, 1.62]  0.05 0.820 | -0.12 [-1.35, 1.11]  -0.04 0.845 | 718.17 | 0.62 | 0.19 | 0.52 |
| **Depression (HADS-D)** | -0.13 [-1.77, 1-51]  -0.04 0.873 | 791.03 | 0.50 | 0.07 | 0.44 | -0.11 [-1.80, 1.57]  -0.03 0.893 | 0.67 [-2.04, 0.70]  -0.20 0.322 | 749.45 | 0.52 | 0.10 | 0.47 |
| **Quality of life (SF12)**  *Physical* | -0.04 [-3.88, 3.80]  0.00 0.983 | 1011.62 | 0.35 | 0.04 | 0.32 | 1.10 [-2.67, 4.87]  0.19 0.562 | 1.23 [-0.73, 3.19]  0.22 0.215 | 921.92 | 0.05 | 0.04 | 0.03 |
| *Mental* | 0.86 [-2.82, 4.54]  0.15 0.643 | 956.31 | 0.03 | 0.02 | 0.01 | 1.10 [-2.67, 4.87]  0.19 0.652 | 1.23 [-0.73, 3.19]  0.22 0.215 | 921 | 0.05 | 0.04 | 0.02 |

**Supplementary *Table 3.*** *PsicoCare: Prediction models coefficients and inferential statistics for psychological outcomes*

|  | **M1** |  |  |  |  | **M2** |  |  |  |  |  |
| --- | --- | --- | --- | --- | --- | --- | --- | --- | --- | --- | --- |
| **Predicted value** | **b 95% CI**  ***β* p-value** | **AICc** | **R^2^**  **Conditional** | **R^2^**  **Marginal** | **icc** | **b_treatment_** **95% CI**  ***β* p-value** | **biam**  **95% CI**  ***β* p-value** | **AICc** | **R^2^**  **Conditional** | **R^2^**  **Marginal** | **icc** |
| **Anxiety (HADS-A)** | -1.46 [-3.07, 0.15]  -0.42 0.0759 | 678.31 | 0.59 | 0.01 | 0.58 | 0.33 [-2.27, 2.95]  0.10 0.79545 | -2.75 [-6.27, 0.76]  -0.79 0.12221 | 623.77 | 0.59 | 0.24 | 0.45 |
| **Depression (HADS-D)** | -2.08 [-3.9, -0.26]  -0.61 0.02541 | 680.57 | 0.47 | 0.03 | 0.45 | -0.59 [-3.54, 2.35]  -0.17 0.68842 | -1.68 [-5.64, 2.27]  -0.49 0.39673 | 636.96 | 0.48 | 0.12 | 0.41 |
| **Anger (STAXI 2)**  *Anger trait* | -2.50 [-4.65, -0.34]  -0.48 0.02335 | 780.93 | 0.69 | 0.02 | 0.68 | -1.78 [-4.99, 1.42] –  0.34 0.26812 | -1.34 [-5.68, 2.98]  -0.26 0.53557 | 715.42 | 0.73 | 0.14 | 0.69 |
| *Anger-out* | -0.62 [-2.06, 0.82]  -0.20 0.39421 | 659.27 | 0.60 | 0.01 | 0.60 | -0.23 [-2.54, 2.08]  -0.07 0.83997 | 0.18 [-2.94, 3.31]  0.06 0.90521 | 622.47 | 0.61 | 0.07 | 0.58 |
| *Anger-in* | -2.03 [-3.52, -0.55]  -0.65 0.00753 | 658.32 | 0.57 | 0.03 | 0.55 | -0.64 [-3.02, 1.72]  -0.21 0.5859 | -2.12 [-5.33, 1.09]  -0.68 0.19134 | 619.24 | 0.57 | 0.10 | 0.52 |
| *Anger control-out* | -0.67 [-2.4, 1.06]  -0.17 0.44397 | 714.37 | 0.65 | 0.04 | 0.63 | -3.20 [-5.90, -0.51]  -0.79 0.02062 | 4.25 [0.61, 7.90]  1.06 0.02288 | 669.04 | 0.68 | 0.09 | 0.65 |
| *Anger control-in* | -0.71 [-3.06, 1.65]  -0.16 0.55213 | 760.24 | 0.47 | 0.03 | 0.45 | -3.7 [-7.21, -0.19]  -0.82 0.03904 | 5.71 [0.95, 10.47]  1.27 0.01947 | 699.84 | 0.55 | 0.16 | 0.47 |
| **Coping (COPE 28)**  *Cognitive* | 2.33 [0.26, 4.4]  0.56 0.02771 | 734.46 | 0.53 | 0.07 | 0.50 | 2.02 [-1.32, 5.36]  0.47 0.23153 | 0.39 [-4.14, 4.93]  0.09 0.86232 | 689.08 | 0.54 | 0.14 | 0.47 |
| *Avoidance* | 0.54 [-1.7, 2.78]  0.13 0.63518 | 671.20 | 0.49 | 0.01 | 0.49 | 0.75 [-2.92, 4.42]  0.17 0.68219 | 0.13 [-4.82, 5.08]  0.03 0.95808 | 619.63 | 0.50 | 0.21 | 0.37 |
| *Social support* | 1.67 [0.05, 3.29]  0.49 0.04332 | 681.49 | 0.58 | 0.05 | 0.55 | 1.09 [-1.53, 3.71]  0.31 0.40757 | 0.67 [-2.88, 4.22]  0.19 0.70557 | 637.25 | 0.59 | 0.19 | 0.49 |
| *Spiritual* | 0.85 [0.18, 1.52]  0.56 0.01326 | 464.87 | 0.63 | 0.05 | 0.61 | 1.64 [0.6, 2.68]  1.05 0.00255 | -1.34 [-2.75, 0.07]  -0.86 0.06138 | 448.24 | 0.67 | 0.15 | 0.61 |
| **Emotional regulation (DERS)** | -5.36 [-13.31, 2.6]  -0.31 0.18526 | 1100.43 | 0.62 | 0.01 | 0.61 | 5.59 [-6.81, 17.99]  0.33 0.37011 | -15.73 [-32.53, 1.06]  -0.93 0.06572 | 996.39 | 0.61 | 0.11 | 0.57 |
| *Lack of emotional attention* | 1.14 [-0.63, 2.9]  0.36 0.20485 | 678.02 | 0.41 | 0.03 | 0.40 | 3.03 [0.28, 5.78]  0.95 0.03121 | -3.60 [-7.33, 0.13]  -1.13 0.05835 | 637.12 | 0.45 | 0.08 | 0.41 |
| *Emotional confusion* | -0.07 [-1.29, 1.15]  –0.02 0.91411 | 636.68 | 0.69 | 0.02 | 0.69 | 1.01 [-0.9, 2.94]  0.34 0.29364 | -1.37 [-3.98, 1.23]  -0.46 0.29489 | 590.90 | 0.70 | 0.18 | 0.64 |
| *Emotional rejection* | -2.69 [-5.48, 0.1]  -0.42 0.05844 | 840.09 | 0.66 | 0.01 | 0.65 | 0.60 [-3.85, 5.05]  0.10 0.78808 | -5.00 [-11.03, 1.02]  -0.80 0.1015 | 777.91 | 0.65 | 0.05 | 0.63 |
| *Emotional lack of control* | -2.20 [-5.34, 0.94]  –0.34 0.16882 | 849.20 | 0.55 | 0.01 | 0.54 | 1.48 [-3.45, 6.42]  0.24 0.54875 | -4.70 [-11.4, 1.99]  -0.77 0.16474 | 772.41 | 0.51 | 0.13 | 0.44 |
| *Life interference* | -1.49 [-3.4, 0.42]  -0.44 0.12426 | 692.79 | 0.37 | 0.03 | 0.36 | -0.12 [-3.03, 2.79]  -0.04 0.93287 | -1.50 [-5.46, 2.45]  -0.46 0.44903 | 644.28 | 0.40 | 0.07 | 0.36 |
| **Dispositional optimism (LOT-R)** | 1.02 [-0.77, 2.82]  0.25 0.2597 | 723.72 | 0.64 | 0.01 | 0.64 | 0.37 [-2.48, 3.22]  0.09 0.79514 | 0.99 [-2.87, 4.85]  0.24 0.60894 | 669.11 | 0.63 | 0.13 | 0.58 |
| **Psychological strengths (PERMA)** | 0.46 [-0.36, 1.28]  0.29 0.27231 | 494.34 | 0.49 | 0.03 | 0.47 | -0.43 [-1.7, 0.83]  -0.27 0.49339 | 1.37 [-0.35, 3.09]  0.86 0.11599 | 465.50 | 0.53 | 0.18 | 0.42 |
| *Achievement* | 0.73 [-0.1, 1.56]  0.44 0.08275 | 503.60 | 0.54 | 0.05 | 0.52 | -0.19 [-1.42, 1.03]  -0.12 0.75179 | 1.33 [-0.32, 3]  0.79 0.11211 | 469.82 | 0.62 | 0.22 | 0.51 |
| *Engagement* | 0.19 [-0.73, 1.11]  .11 0.67928 | 514.67 | 0.42 | 0.01 | 0.42 | -0.18 [-1.61, 1.24]  -0.11 0.79618 | 0.45 [-1.48, 2.39]  0.27 0.6373 | 491.04 | 0.47 | 0.11 | 0.41 |
| *Meaning* | 0.66 [-0.2, 1.52]  0.38 0.13272 | 514.87 | 0.53 | 0.02 | 0.52 | -0.57 [-1.89, 0.74]  -0.33 0.38674 | 1.94 [0.15, 3.73]  1.12 0.03396 | 486.90 | 0.59 | 0.15 | 0.52 |
| *Positive Emotions* | 0.65 [-0.33, 1.64]  0.35 0.19262 | 538.83 | 0.48 | 0.04 | 0.46 | -0.58 [-2.08, 0.92]  -0.31 0.43996 | 1.93 [-0.1, 3.96]  1.02 0.06183 | 507.17 | 0.54 | 0.15 | 0.45 |
| *Social Relationships* | 0.20 [-0.72, 1.12]  0.11 0.66886 | 528.94 | 0.52 | 0.02 | 0.51 | -0.35 [-1.83, 1.12]  -0.19 0.63032 | 0.88 [-1.11, 2.89]  0.49 0.3766 | 495.97 | 0.51 | 0.19 | 0.39 |
| **Quality of life (SF12)**  *Physical* | 2.16 [-1.5, 5.83]  0.33 0.24456 | 863.66 | 0.40 | 0.01 | 0.39 | 0.22 [-5.55, 6]  0.03 0.9373 | 3.55 [-4.28, 11.38]  0.54 0.36783 | 810.11 | 0.43 | 0.05 | 0.40 |
| *Mental* | 1.98 [-1.64, 5.61]  0.37 0.28116 | 819.34 | 0.08 | 0.03 | 0.05 | 3.07 [-2.78, 8.93]  0.56 0.2975 | -0.94 [-8.93, 7.04]  -0.17 0.81375 | 772.97 | 0.09 | 0.07 | 0.01 |

**Supplementary *Table 4****. PsicoCare: Prediction models coefficients and inferential statistics for ergometry outcomes*

|  | **M1** |  |  |  |  |
| --- | --- | --- | --- | --- | --- |
| **Predicted value** | **b 95% CI**  ***β* p-value** | **AICc** | **R^2^**  **Conditional** | **R^2^**  **Marginal** | **ICC** |
| **METS** | 0.36 [-1.53, 2.26]  0.09 0.703 | 649.96 | 0.66 | 0.03 | 0.65 |
| **Total time on ergometry** | 0.24 [1.59, 2.07]  0.06 0.795 | 625.58 | 0.69 | 0.04 | 0.67 |
| **Heart rate**  *Maximum* | 2.51 [-14.75, 19.78]  0.09 0.773 | 1114.33 | 0.31 | 0.01 | 0.30 |
| *Resting* | 2.08 [-8.49, 12.64]  0.12 0.697 | 990.16 | 0.46 | 0.00 | 0.46 |
| **Systolic blood pressure (mmHG)**  *Maximum* | -0.30 [-19.35, 18.74]  -0.01 0.974 | 1143.16 | 0.49 | 0.07 | 0.45 |
| *Resting* | -3.48 [-20.49, 13.53]  -0.14 0.685 | 1043.94 | 0.30 | 0.04 | 0.27 |
| **Diastolic blood pressure (mmHG)** | -0.48 [-11.06, 10.09]  -0.03 0.927 | 994.01 | 0.34 | 0.02 | 0.33 |
| *Maximum* |  |  |  |  |  |
| *Resting* | -6.35 [-16.43, 3.73]  -0.44 0.214 | 922.93 | 0.24 | 0.04 | 0.21 |
| **Bruce value*** | -2.18 [-4.00, -0.36]   0.019 | 144.11 | 0.15 | 0.13 | 0.03 |
| **Clinical significance*** | -2.00 [-8.07, 4.07]  0.518 | 101.87 | 0.99 | 0.02 | 0.97 |
| **Electrical significance*** | 3.81 [-9.78, 2.16]  0.211 | 63.69 | 0.98 | 0.01 | 0.98 |
| **Arrythmia presence during ergometry*** | --- | --- | --- | --- | --- |

*Note: *, denote the use of Binomial link on regression, therefore coefficients are expressed as log odds*

**Supplementary *Table 5.*** *PsicoCare: Prediction models coefficients and inferential statistics for biochemical and anthropometrical outcomes*

|  | **M1** |  |  |  |  |
| --- | --- | --- | --- | --- | --- |
| **Predicted value** | **b 95% CI**  ***β* p-value** | **AICc** | **R2**  **Conditional** | **R2**  **Marginal** | **ICC** |
| **Heart rate** | 6.09 [-3.82, 16.01]  0.39 0.244 | 993.68 | 0.46 | 0.09 | 0.40 |
| **Systolic blood pressure (mmHG)** | -18.86 [-45.16, 7.45]  -0.51 0.158 | 1291.64 | 0.08 | 0.03 | 0.04 |
| **Diastolic blood pressure (mmHG)** | 1.19 [-7.16, 9.54]  0.10 0.776 | 1005.43 | 0.12 | 0.02 | 0.11 |
| **Ldl-cholesterol (mg/dl)** | -15.27 [39.50, 8.96]  -0.39 0.213 | 1275.23 | 0.28 | 0.22 | 0.07 |
| **Glycated hemoglobin** | -0.13 [-0.79, 0.53]  0.11 0.696 | 355.76 | 0.65 | 0.07 | 0.62 |
| **BMI*** | -0.04 [-8.68, 8.59]  0.00 0.992 | 763.4 | 0.06 | - | - |

**Note:** asterisk denote results from a regression without random effect due to singularity effects from low variance components

**Supplemental Material 4:** *Health Pills efficacy data.*

**Supplementary Table 6.** *Health Pills: Psychological outcomes descriptive statistics and change score between time 1 and time 2 assessment*

|  | **Control Group [M (SD)]** | | | ***PsicoCare* Group [M (SD)]** | | |
| --- | --- | --- | --- | --- | --- | --- |
| **Measure** | *Pretreatment (T1)* | *Posttreatment (T2)* | *Change Score (Δ)* | *Pretreatment (T1)* | *Posttreatment (T2)* | *Change Score (Δ)* |
| **Anxiety (HADS-A)** | 8.5 (3.5) | 7.3 (3.5) | 1.0 (3.1) | 9.2 (2.9) | 8.1 (3.2) | 1.4 (3.1) |
| **Depression (HADS-D)** | 4.1 (3.1) | 3.0 (3.0) | 0.7 (2.9) | 5.3 (3.5) | 4.5 (3.5) | 1.2 (3.8) |
| **Quality of life (SF12)**  *Physical*  *Mental* | 41.7 (7.9)  47.0 (5.6) | 41.6 (6.5)  47.3 (5.6) | 1.1 (7.7)  -0.9 (7.6) | 42.4 (6.9)  45.7 (5.9) | 41.8 (6.6)  46.9 (5.1) | 0.1 (8.5)  -1.4 (7.9) |
